# In-Treatment Kinetics of Peripheral Blood Immune Markers in PD-L1 High Non-Small Cell Lung Cancer and Prognostic Relevance for Immunotherapy Outcomes

**DOI:** 10.3390/cancers18101623

**Published:** 2026-05-17

**Authors:** Ioannis P. Trontzas, Ioanna-Evdokia Galani, Emmanouil Panagiotou, Efthymia Theofani, Anastasia Georganta, Konstantinos G. Kyriakoulis, Anastasia Palaiologou, Ioannis Vathiotis, Constantin Tamvakopoulos, Evangelos Andreakos, Konstantinos N. Syrigos

**Affiliations:** 1 3rd Department of Medicine, ‘Sotiria’ Hospital of Respiratory Diseases, National and Kapodistrian University of Athens, 152 Mesogion Ave., 11527 Athens, Greecekonkyriakoulis@gmail.com (K.G.K.);; 2 Laboratory of Immunology, Center for Clinical, Experimental Surgery and Translational Research, Biomedical Research Foundation of the Academy of Athens (BRFAA), 4 Soranou Ephessiou Street, 11527 Athens, Greece; igalani@bioacademy.gr (I.-E.G.);; 3 Division of Pharmacology-Pharmacotechnology, Center for Clinical, Experimental Surgery and Translational Research, Biomedical Research Foundation of the Academy of Athens (BRFAA), 4 Soranou Ephessiou Street, 11527 Athens, Greecectamvakop@bioacademy.gr (C.T.)

**Keywords:** non-small cell lung cancer, immunotherapy, peripheral blood immune markers, CRP, complement protein C4, IL-6

## Abstract

**Background:** A substantial proportion of patients with non-small cell lung cancer (NSCLC) derive limited clinical benefit from immunotherapy. Monitoring of peripheral blood immune markers (PBIMs) may emerge as a useful tool to predict treatment outcomes with immune checkpoint inhibitors (ICIs). **Patients/Methods:** We prospectively measured several PBIMs in a PD-L1 high (TPS ≥ 50%) NSCLC cohort of patients treated with first-line pembrolizumab monotherapy. Kinetics over the first year of treatment were assessed at baseline (T0) and at 21 days (T1), 3 months (T2), 6 months (T3) and at 1 year (T4) post-treatment initiation. Associations with clinical outcomes were explored after a 2-year follow-up period. **Results:** In total, 31 patients with PD-L1 high locally advanced or metastatic NSCLC were prospectively enrolled. Over the first year of treatment, levels of CRP, IL-17α, IL-6, and IL-8 were significantly decreased. Early kinetics analysis showed significant decrease in total leukocytes, neutrophils, CRP, and MIP-3α/CCL20, as well as significant transient elevation of ITAC/CXCL11, IL-1β, IL-7, and TNFα, during the first 3 months of treatment. Early percent changes (Δ% at T1 and at T2) of ‘low’ vs. ‘high’ pretreatment levels showed significant differences for LDH, ITAC/CXCL11, GM-CSF, MIP-1α/CCL3, IL-2, IL-4, IL-5, and sPD-L1. Longitudinal analysis, stratified per responders and for pre-progression fluctuations, did not reveal significant findings. Among markers with acceptable discriminative performance, higher baseline CRP, complement C4, and IL-6 levels were associated with poorer clinical outcomes. In multivariable analysis, only C4 retained independent prognostic significance; however, integration of these PBIMs into composite indices improved prognostic performance. **Conclusions:** In this prospective study, longitudinal monitoring of PBIMs provided descriptive insights into immune and inflammatory dynamics during pembrolizumab treatment; however, no significant associations were observed between in-treatment biomarker kinetics and clinical outcomes. In exploratory analyses, baseline CRP, complement C4, and IL-6 levels were associated with clinical outcomes, and their integration into composite indices improved prognostic performance. These findings suggest that specific baseline PBIMs may carry prognostic relevance, while the role of in-treatment monitoring remains to be further clarified in larger prospective studies.

## Simple Summary

Immunotherapy has improved outcomes for patients with advanced lung cancer, but many patients do not benefit or eventually stop responding. There is a need for simple and reliable ways to predict who will respond to treatment. In this study, we monitored blood-based immune markers in patients receiving immunotherapy over time using routine blood samples. We observed that several markers related to inflammation decreased during treatment, while others showed early temporary increases, reflecting activation of the immune system. Importantly, higher levels of certain markers before treatment, particularly a protein of the complement system (C4), as well as C-reactive protein and interleukin-6, were associated with poor outcomes. Combining these markers improved their predictive ability. These findings suggest that simple blood tests may help track how the immune system responds to treatment and could support better patient monitoring and treatment decisions in the future.

## 1. Introduction

Immunotherapy with immune checkpoint inhibitors (ICIs) has revolutionized the management of metastatic non-oncogene-addicted non-small cell lung cancer (NSCLC). In a disease setting with poor prognosis, ICIs offer significant improvements, with a reported median overall survival ranging from 17 to 26 months in the randomized trials, with or without the addition of chemotherapy [1]. Treatment benefit appears to be PD-L1-dependent, with PD-L1 high patients (tumor proportion score, TPS ≥ 50%) demonstrating the most favorable responses, leading to the respective regulatory approval of anti-PD-1 monotherapy in the first-line setting [2]. Long-term benefits are also more pronounced in PD-L1 high patients, with 5-year survival rates of up to 32% in the overall population and with approximately 82% of those completing the 2-year regimen still alive after 5 years [3]. Despite these unprecedented advances, most patients do not derive meaningful clinical benefits. Many patients exhibit primary resistance, defined by the Society for Immunotherapy of Cancer (SITC) consensus as progressive disease (PD) within 6 months of treatment despite adequate drug exposure (typically at least two cycles or 6 weeks of treatment) [4], and, even among initial responders, a substantial proportion eventually develop acquired resistance (defined as PD following 6 months of treatment after an initially documented objective response or stable disease) [4,5]. The current limitations of immunotherapy warrant the need for exploration of more precise biomarkers.

While tissue-based immune markers among others have been thoroughly studied (e.g., PD-L1, tumor-infiltrating lymphocytes (TILs), regulatory T-cells (Tregs), cancer-associated fibroblasts (CAFs), etc.) in NSCLC, few have entered clinical practice due to several inherent limitations [6]. Firstly, tissue-based markers provide a temporary impression of the tumor immune microenvironment (TIME), which is continuously reshaping during treatment. In addition, the monitoring of tumor–immune system interactions would require several tissue samplings, which would not be feasible due to challenges associated with multiple biopsies in cancer patients. Furthermore, biopsy specimens provide only a small fragment of the tumor’s immune landscape within a rather spatially heterogenous TIME. Lastly, it appears that the assessment of isolated immune markers does not fully capture the complexity of immune responses [6]. Incorporation of newer technological developments on digital pathology, coupled with multi-omics, artificial intelligence, and machine learning tools, may provide more sophisticated composite biomarker profiles [7,8].

An emerging approach for predicting response, forthcoming resistance, and assessing the evolving immune interactions during treatment is the monitoring of peripheral blood immune markers (PBIMs). Peripheral blood monitoring offers a complementary diagnostic tool with minimally invasive, dynamic assessment of the immune system during treatment. The feasibility of this approach has led to the investigation of a variety of immune parameters, including complete blood count (CBC) parameters (e.g., total leukocytes, neutrophil, lymphocyte, platelet counts), lymphocyte subsets, inflammatory markers, complement system proteins, cytokines, soluble immune checkpoint proteins, and others [6]. While none of these have been adopted in the clinical routine, some have been associated with outcomes of checkpoint inhibition in NSCLC [6]. For instance, several lymphocyte ratios have been linked with immunotherapy outcomes, with elevated pre- and post-treatment neutrophil-to-lymphocyte ratios (NLRs) and an elevated pre-treatment platelet-to-lymphocyte ratio (PLR) being associated with worse ICI outcomes, whereas a higher baseline lymphocyte-to-monocyte ratio (LMR) conferred a more favorable profile for immunotherapy in meta-analyses [9,10]. Similarly, elevated levels of inflammatory markers and ratios (e.g., CRP, CRP-to-albumin ratio), as well as parameters reflecting the tumor burden (e.g., LDH), have been associated with worse ICI outcomes [11,12]. Additionally, immunophenotypes reflecting abundant activated peripheral CD8+ cells, low CD4+/CD8+ ratio, and in-treatment elevation of CD8+ peripheral cells have been linked with better ICI outcomes [13,14]. Other peripheral parameters, such as cytokines, peripheral soluble immune checkpoint proteins (e.g., soluble PD-L1 (sPD-L1)), and complement system components (e.g., C3, C4) have also been studied, but their roles remain unclear in immunotherapy for lung cancer [14,15,16,17]. Collectively, most of these studies have recognized an unfavorable effect of higher systemic inflammation on ICI outcomes and have reported distinct immunophenotypes associated with response.

Coupled with pathology and genomic information, the variety of PBIMs could deliver a real-time map of the immune landscape. However, most studies are retrospective and include heterogenous patient populations and treatment regimens, and much less is known regarding the dynamic evolution of these markers during therapy. As a result, more prospective evidence is needed to establish their role.

In this context, prospective data on the longitudinal dynamics of PBMIs during immunotherapy remain limited, particularly in homogenous treatment-naïve cohorts. Moreover, the relationship between early in-treatment immune changes and clinically meaningful outcomes has not been fully elucidated. In the present study, we prospectively evaluated a broad panel of PBIMs at multiple predefined timepoints during first-line pembrolizumab monotherapy in a PD-L1 high NSCLC cohort. We aimed to (i) characterize longitudinal immune and inflammatory kinetics, (ii) explore early dynamic changes during treatment, and (iii) investigate their association with early and longer-term outcomes. By focusing on a uniform population and integrating biomarker domains, this study sought to provide insight into real-time tumor–immune system interactions and to address current gaps in prospective biomarker research. Moreover, treatment with pembrolizumab monotherapy allowed assessment of PBIMs’ complementary role in a cohort characterized by a known, favorable, tissue-based prognostic factor (PD-L1 ≥ 50%).

## 2. Methods

### 2.1. Patients and Treatment

All consecutive patients with locally advanced or metastatic NSCLC, who were followed at the Oncology Unit of the 3rd Department of Internal Medicine, ‘Sotiria’ Hospital of Respiratory Diseases, Athens, Greece, and met all the inclusion and none of the exclusion criteria were prospectively enrolled in the study. Inclusion criteria included: (i) histologically confirmed diagnosis of locally advanced or metastatic NSCLC, (ii) scheduled to receive first-line immunotherapy with pembrolizumab as monotherapy per national guidelines and physician’s choice, (iii) received no prior systemic anti-cancer treatment for at least two months, (iv) aged > 18 years, and (v) provided written informed consent. Exclusion criteria were (i) the administration of other systemic anti-cancer treatment in combination with immunotherapy, or administration of such therapy within less than two months prior to initiation of immunotherapy; and (ii) the administration of immunotherapy prior to patient enrollment in the study.

Eligible patients received first-line pembrolizumab monotherapy. Pembrolizumab was administered at a fixed dose of 200 mg every 21 days for up to two years, or until PD, death (whichever occurred first), or unacceptable toxicity, in accordance with ESMO guidelines [2]. Palliative local treatments (e.g., radiotherapy, cyber-knife, thermal ablation, etc.) before and during systemic immunotherapy were permitted.

### 2.2. Clinical and Laboratory Follow-Up

Baseline clinicopathological characteristics were recorded at study entry. Clinical and radiological response was assessed every three months, or earlier at the discretion of the treating physician, according to RECIST version 1.1 [18]. Immune-related adverse events (irAEs) were documented at each scheduled treatment visit, in accordance with the Common Terminology Criteria for Adverse Events (CTCAE), version 5.0 [19], and managed according to ESMO guidelines [20]. Clinical follow-up continued for two years or was discontinued earlier in the event of PD, severe irAE, or any other reason for treatment discontinuation and/or initiation of second-line therapy.

Baseline clinicopathological characteristics included: age, sex, Eastern Cooperative Oncology Group performance status (ECOG PS), smoking history, disease stage and histological type, PD-L1 expression (TPS, %), presence of driver mutations, palliative/local radiotherapy (during treatment or within the last two months), and administration of corticosteroids or other immunomodulatory agents.

Laboratory monitoring of PBIMs was performed at the following timepoints: before the first pembrolizumab infusion (baseline, T0), before the second cycle (21 days, T1), at three months (T2), at six months (T3), and at one year (T4). Clinical and laboratory follow-up schedules are illustrated in Supplementary Figure S1.

### 2.3. Laboratory Procedures and Analyses

In total, 30 mL of blood were collected at each sampling, of which 10 mL were split for biochemical analysis as serum and for CBC as plasma (EDTA tubes). The remaining 20 mL was centrifuged at 1800 rpm (≈290× *g*) at 19–20 °C for 20 min in a refrigerated centrifuge (S300TRH, KUBOTA Corporation, Tokyo, Japan), and then used as plasma for the analysis of cytokines and soluble PD-L1 (sPD-L1). Centrifuged plasma samples were stored in −80 °C for up to three months before laboratory analysis.

#### 2.3.1. Assessment of Cell Counts and Biochemical Parameters

The assessment of cell counts (total leukocytes; leukocyte subtypes—neutrophils, lymphocytes, and monocytes; and platelet counts) from CBC and of biochemical parameters (CRP, complement proteins C3 and C4, LDH, and albumin) was performed at the institutional laboratory based in ‘Sotiria’ Hospital as part of the routine patient evaluation prior to treatment. In addition to PBIMs, LDH was also analyzed as an indicator of tumor burden, as well as albumin as a constitutional factor reflecting the nutritional status, for incorporation in composite indices along with immune markers.

Based on the measured absolute values, the derived leukocyte ratios (NLR, derived NLR (dNLR), LMR, PLR), biochemical ratios (complement ratio C3/C4, CAR), and composite indices, such as the Lung Immune Prognostic Index (LIPI) and the Systemic Inflammation Index (SII), previously associated with prognosis in immunotherapy [9,10,12,21,22] were calculated.

The dNLR was calculated using the following formula (absolute values derived from the baseline cell counts):

$$\mathrm{dNLR}\;=\;\frac{Absolute\;neutrophil\;count}{Absolute\;leukocyte\;count\;-\;Absolute\;neutrophil\;count}.$$

The LIPI score was calculated for each patient based on two parameters: dNLR > 3 and LDH > ULN (upper normal limit, set at 248 IU/L for the local laboratory). Each positive parameter corresponded to 1 point, resulting in a total score ranging from 0 to 2. Patients were classified into three groups: good prognosis (0), intermediate prognosis (1), and poor prognosis (2).

The SII was calculated as follows (absolute values derived from the baseline cell counts):

$$\mathrm{SII}\;=\;\frac{Absolute\;platelet\;count\;\times \;Absolute\;neutrophil\;count}{Absolute\;lymphocyte\;count}.$$

#### 2.3.2. Cytokine Analysis

Frozen plasma samples were used for cytokine analysis with the MILLIPLEX^®^ MAP Human High Sensitivity T Cell Panel (#HSTCMAG-28SK, Merck Millipore, Burlington, MA, USA). This method is a bead-based multiplex immunoassay specifically designed for the Luminex^®^ xMAP^®^ platform (Luminex Corporation, Austin, TX, USA), which enables the simultaneous quantification of 21 cytokines and chemokines related with T-cell responses, as well as with innate immune biology and/or neutrophil-related inflammation. The cytokines/chemokines analyzed in the panel were: ITAC/CXCL11, GM-CSF, Fractalkine/CX3CL1, IFN-γ, IL-10, MIP-3α/CCL20, IL-12/p70, IL-13, IL-17α/CTLA-8, IL-1β, IL-2, IL-21, IL-4, IL-5, IL-23, IL-6, IL-7, IL-8/CXCL8, MIP-1α/CCL3, MIP-1β/CCL4, and TNFα. Thawed plasma samples were centrifuged at 13,000 rpm (≈15,000× *g*) for 10 min at 4 °C immediately before analysis. Each analysis was performed according to the manufacturer’s protocol for plasma samples, using the recommended dilutions and standard curve concentrations. Samples were analyzed on a Luminex 200 system with Luminex xPonent v.3.1 software, according the manufacturer’s instructions (Merck Millipore). The assay sensitivity ranged from 0.11 to 8.17 pg/mL, depending on the analyte. The analyzed cytokines and the lower limits of detection (LOD, pg/mL) are summarized in Supplementary Table S1. For data analyses, lower included values were set at the LOD of each cytokine.

#### 2.3.3. Measurement of Soluble PD-L1 Levels

Concentrations of sPD-L1 were measured using the Human PD-L1 ELISA Kit (#BMS2327, Thermo Fisher Scientific, formerly Life Technologies, Waltham, MA, USA). This method is an enzyme-linked immunosorbent assay (ELISA) designed for the detection of human PD-L1 (CD274) protein in plasma, serum, cell lysates, and cell culture supernatants. Each analysis was performed according to the manufacturer’s instructions. Data analysis was performed with QuantAssay software. For each assay, a standard curve was generated in the range of 4.7–300 pg/mL, with an LOD of 0.6 pg/mL. All samples were analyzed in duplicate, and the mean value was reported. Samples exceeding the linear range were appropriately diluted and re-analyzed. Values below the LOD were included in the analyses at the cutoff of LOD.

Cytokine analyses and measurements of sPD-L1 were performed in collaboration with the Laboratory of Immunology, Center for Clinical, Experimental Surgery and Translational Research, Biomedical Research Foundation of the Academy of Athens (BRFAA), Greece.

### 2.4. Study Endpoints

The primary endpoints of the study were the dynamic quantification of PBIMs in the subgroup of PD-L1 high patients with metastatic NSCLC during pembrolizumab monotherapy, as well as the evaluation of the kinetics of each parameter at pre-specified timepoints.

Exploratory associations of the baseline levels and of the significant dynamic changes in PBIMs with therapy outcomes were sought as a secondary exploratory study endpoint.

### 2.5. Treatment Outcomes

The association of PBIMs with treatment outcomes was assessed using the following clinical endpoints with the intention to explore potential predictors of early and sustained benefit to immunotherapy:

Clinical benefit at 6 months (CB6)

According to the RECIST (v.1.1) [18], response was categorized as complete response (CR), partial response (PR), stable disease (SD), and PD. Assessment of PD was also based on the treating physician’s clinical judgment. Clinical benefit was defined as the proportion of patients achieving CR, PR, or SD. This reflects the overall percentage of patients who achieve disease stabilization or improvement (disease control rate), thereby highlighting the overall clinical benefit of the therapeutic intervention. For this study, the 6-month clinical benefit (CB6) was used. CB6 was chosen as an exploratory endpoint reflecting early treatment benefit.

Progression-free survival at 2 years (2y PFS)

For this study, considering that follow-up was completed at two years, progression-free survival at 2 years (2y PFS) was used as a clinical endpoint to reflect later and sustained treatment benefit.

### 2.6. Statistical Analysis

Statistical analysis was performed using GraphPad Prism software, version 10.4.1 (GraphPad Software, La Jolla, CA, USA). Baseline characteristics and clinical outcomes were summarized and reported as percentages for categorical variables, and as mean values with standard deviation (SD) or median with interquartile range (IQR), depending on the distribution of continuous variables. The Shapiro–Wilk test was used to assess the normality of data distribution. Probabilities of 2y PFS and 2-year survival were estimated using the Kaplan–Meier method.

The longitudinal dynamics of PBIMs at the pre-specified timepoints were evaluated using the non-parametric Kruskal–Wallis test. Early dynamics, defined as changes between T0–T1 and T0–T2, were also assessed with the non-parametric Wilcoxon test for paired data, including only patients with available paired measurements. In addition, percent changes in early kinetics (Δ%, T0–T1 and T0–T2) were estimated to assess the biological patterns in patients with extreme immunological or inflammatory profiles at baseline. Patients were categorized into ‘high’ and ‘low’ baseline groups according to the median of each parameter. The mean Δ% was then calculated within each group, and groups were compared using the non-parametric Mann–Whitney test.

The Δ% between T0 and T1 or T2 was calculated as:

$$\Delta \%\;=\;\frac{\left(Value\;at\;T1\;or\;T2\;-\;Value\;at\;T0\right)}{Value\;at\;T0}\times 100.$$

The Δ% was also used to compare differences in early kinetics (T0–T1 and T0–T2) between ‘responders’ and ‘non-responders’ (patients achieving CR or PR vs. patients with best response SD or PD). Comparisons of Δ% between the two groups were performed using the Mann–Whitney test. Furthermore, measurements immediately prior to PD were analyzed in patients with available samples. Patients with a sample taken more than three months before PD were excluded. For each patient, the immediately preceding measurement served as the reference value. Comparisons between reference values and pre-PD values were performed using the Mann–Whitney test.

Next, a three-tiered statistical filtering process was applied to explore potentially meaningful prognostic PBIMs for each clinical endpoint. At first, univariate logistic regression analysis was performed between continuous parameters (or, in certain cases, dichotomized categories) and binary CB6 outcomes (benefit vs. PD). For 2y PFS, univariate Cox regression analysis was similarly applied (association of parameters with PD vs. non-PD). The discriminative performance of PBIMs in univariate logistic models was assessed with receiver operating characteristic (ROC) curve analysis using the area under the curve (AUC), while in Cox analyses, it was evaluated with Harrell’s concordance index (C-index). Following identification of parameters with acceptable statistically significant discriminatory values (variables with AUC/C-index > 0.70 and a 95% confidence interval (95%CI) lower bound > 0.5), odds ratios (OR) and hazard ratios (HR) were further assessed. The cutoff value of 0.7 for discriminatory metrics was applied based on the current literature for ‘acceptable to good performance for values between 0.7–0.8′, accounting for the small sample size [23]. Parameters with statistically significant associations but excessively wide CIs (95%CI upper-to-lower ratio ≥ 3) were excluded from further analyses. Biomarkers with acceptable AUC/C-index being significant prognosticators in the univariate analysis were further explored in multivariable logistic regression models, along with ECOG PS and the presence of brain metastases and ‘distant metastases’ (defined as number of organs involved), to assess their prognostic significance when adjusted for established clinical prognosticators. Moreover, a composite score incorporating all significant parameters was synthesized. Each parameter was dichotomized at its median (‘low’ vs. ‘high’), assigned a score of 0 or 1, and summed to generate a composite score per patient. The resulting composite score was independently tested in multivariable models. Finally, to assess the potential confounding effect of baseline corticosteroid/immunomodulatory treatment and recent radiotherapy exposure, exploratory sensitivity analyses were performed for the principal prognostic PBIMs.

Statistical significance was set as two-sided *α* < 0.05 for all analyses.

Values below the LOD were treated as semi-quantitative measurements, as the assays provided continuous numerical estimates even at low concentrations. While these values may have reduced precision, they were considered informative for relative comparisons and were therefore retained in analyses. Absolute values are provided in Supplementary Materials.

## 3. Results

### 3.1. Patients’ Enrollment, Baseline Characteristics, and Treatment Outcomes

Within a two-year period (October 2020 to November 2022), 31 consecutive patients were prospectively enrolled in the study. The current cohort has been previously introduced, and the baseline characteristics and therapeutic outcomes have been reported [24].

In brief, the median age was 72 years (IQR, 53–83), and most patients were males (n = 23, 74.2%). All patients had a histologically confirmed diagnosis of NSCLC, 22 (71.0%) of adenocarcinoma and nine (29.0%) of squamous cell carcinoma. Most patients presented with stage IV disease at treatment initiation (n = 27, 87.1%), while the remainder had stage III disease (n = 4, 12.9%). All patients had PD-L1 high disease (TPS ≥ 50%) with median PD-L1 expression being 80% (50–100). Two patients (6.5%) had tumors with actionable mutations (both KRAS G12C). Most patients presented with ECOG PS of 0 and 1 (n = 11, 35.5% for each group), while the remainder with PS ≥ 2 (n = 9, 29.0%). All patients were either former (n = 22, 71.0%) or current (n = 9, 29.0%) smokers, with a smoking history of 70 pack–years (10–150). Most patients (n = 18, 58.0%) received palliative/local radiotherapy at baseline or within the preceding two months. Radiotherapy was directed to the brain and thorax (n = 7, 22.6% each), while four patients (12.9%) received palliative radiotherapy at multiple sites. Finally, 14 patients (45.2%) were receiving corticosteroids and/or other immunomodulatory agents at study entry. Baseline characteristics are summarized in Supplementary Table S2.

Regarding clinical outcomes, the best objective response was CR in one patient (3.2%), PR in 15 patients (48.4%), SD in seven patients (22.6%), and PD in eight patients (25.8%). Most objective responses (CR, PR) were recorded at three months (n = 14, 45.2%), with the remainder occurring at six months (n = 2, 6.5%). The objective response rate (ORR) of the cohort was 51.6% (n = 16), while 12 patients (38.7%) maintained a stable response at the end of the two-year follow-up period. The clinical benefit rate was 61.3% (n = 19) at six months, 54.8% (n = 17) at one year, and 45.2% (n = 14) at the end of follow-up (two years). In total, 21 patients (67.7%) were alive at the end of follow-up. Median 2y PFS was 17.9 months, while median 2-year survival was not reached. Survival results are summarized in Supplementary Table S3.

Regarding toxicity, most patients experienced at least one irAE (n = 25, 80.6%), and in most cases, more than one organ system was affected (n = 18, 58.0%). Overall, 10 patients (32.3%) required temporary treatment interruption due to irAEs, and in six cases (19.4%) treatment was permanently discontinued. One death due to pneumonitis was recorded (3.2%).

### 3.2. Treatment Administration and Sample Collection

Pembrolizumab was administered every three weeks, according to clinical practice, and peripheral blood sample collection was scheduled at five pre-specified timepoints as discussed earlier. The administration schedule and sampling dates are summarized in Table 1.

At T0, 31 samples were collected, while at subsequent timepoints 30 (T1), 27 (T2), 21 (T3), and 20 (T4) samples were obtained. With regard to pembrolizumab administration, by T1 one infusion had been completed, while by T2, T3, and T4, the median number (IQR) of infusions was 4 (2–4), 7 (5–8), and 13 (8–15), respectively.

To assess potential attrition-related bias, baseline characteristics were compared between patients with complete vs. incomplete longitudinal sampling (Supplementary Table S4). No statistically significant imbalances were found between the two groups.

The distribution of treatment administrations reflects the challenges of dynamic treatment monitoring in real-world clinical practice conditions, while the declining number of drawn samples reflects the dropout of patients from the study due to PD or other reason.

### 3.3. Dynamic Changes in Peripheral Blood Immune Markers During Treatment

#### 3.3.1. Kinetics Across All Timepoints

**Figure 1:**
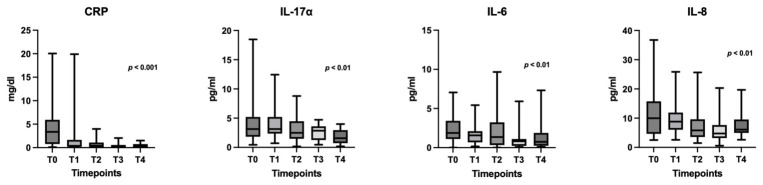
Peripheral blood immune markers demonstrating significant fluctuations during the first year of treatment. Results are presented as box and whisker plots with min/max. Abbreviations: CRP: C-reactive protein; IL: interleukin; T0: pretreatment (baseline); T1: 21 days (before cycle 2); T2: 3 months; T3: 6 months; T4: 1 year.

Comparison of the cell counts did not reveal significant differences (*p* > 0.05) for any parameter during treatment (Supplementary Figure S2). Comparisons of the biochemical parameters showed significant decrease in CRP during treatment (*p* < 0.001) (Figure 1), while the rest did not change significantly (Supplementary Figure S3). Comparison across timepoints showed significant decreases for IL-17α, IL-6, and IL-8 (*p* < 0.01) (Figure 1), and no significant changes for other cytokines (Supplementary Figure S4). No significant fluctuations were seen in the levels of sPD-L1 across all timepoints (Supplementary Figure S4).

#### 3.3.2. Assessment of Early Kinetics (PBIM Fluctuations Between T0–T1 and T0–T2)

**Figure 2:**
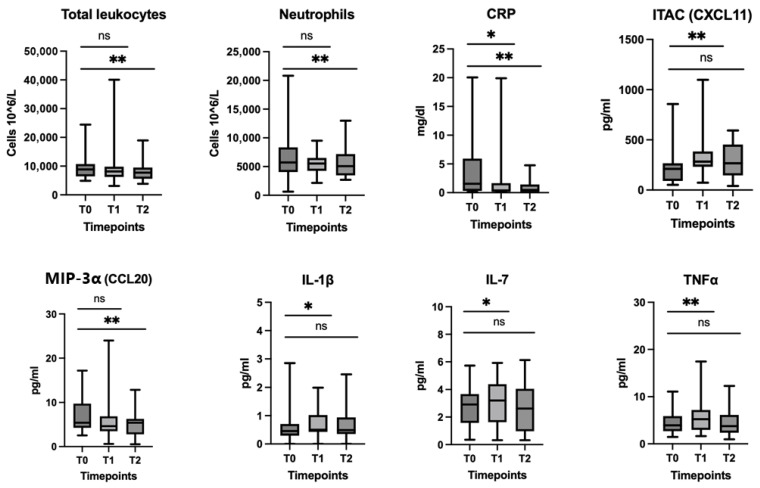
Peripheral blood immune markers with significant fluctuations within the first three months (T0–T1 and T0–T2 kinetics) of treatment. Results are presented as box and whisker plots with min/max. Abbreviations: CCL20, C-C motif chemokine ligand 20; CRP, C-reactive protein; CXCL11, C-X-C motif chemokine ligand 11; ITAC, interferon-inducible T-cell α chemoattractant; IL, interleukin; MIP-3α, macrophage inflammatory protein-α; TNFα, tumor necrosis factor α; T0, pretreatment (baseline); T1, 21 days (before cycle 2); T2, 3 months. * *p* < 0.05; ** *p* < 0.01; ns: *p* > 0.05.

Paired comparisons of cell counts from CBC showed significant decrease in total leukocytes (*p* < 0.05) and neutrophils (*p* < 0.01) between T0 and T2, as well as no significant fluctuations between T0 and T1 (Figure 2). A significant decrease in CRP was shown between T0 and T1 (*p* < 0.05) that was more pronounced at three months (*p* < 0.1) (Figure 2). In cytokine analyses, MIP-3α/CCL20 showed a significant decrease over the first three months of treatment (*p* < 0.01) but not at T1 (Figure 2), whereas ITAC/CXCL11 (*p* < 0.01), IL-1β (*p* < 0.05), IL-7 (*p* < 0.05), and TNFα (*p* < 0.01) were transiently increased at T1, though the fluctuation at T2 was not significant (Figure 2). The remaining PBIMs did not show any significant changes between T0–T1 and T0–T2 (Supplementary Figures S5–S7).

#### 3.3.3. Early Dynamics According to ‘High’ vs. ‘Low’ Pretreatment Values

**Figure 3:**
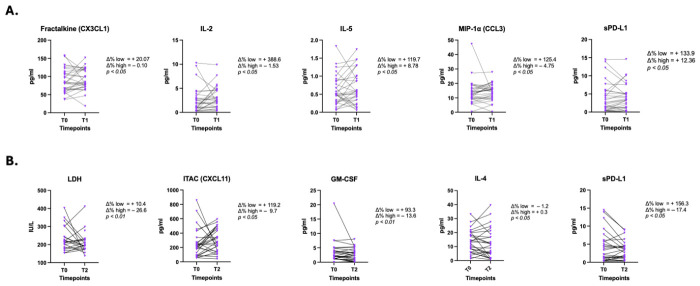
Significant early dynamic changes (Δ%, T0–T1 and T0–T2) between the kinetics of ‘low’ and ‘high’ baseline values of peripheral blood parameters. (**A**) Kinetics between T0 and T1. (**B**) Kinetics between T0 and T2. Abbreviations: CCL3, C-C motif chemokine ligand 3; CXCL11, C-X-C motif chemokine ligand 11; CX3CL1, C-X3-C motif chemokine ligand 1; GM-CSF, granulocyte–macrophage colony-stimulating factor; IL, interleukin; ITAC, interferon-inducible T-cell alpha chemoattractant; LDH, lactate dehydrogenase; MIP-1α, macrophage inflammatory protein-1 α; sPD-L1, Soluble programmed-death ligand-1; T0, pretreatment (baseline); T1, 21 days (before cycle 2); T2, 3 months; Δ%, percent change.

Comparison of Δ% between ‘low’ and ‘high’ pretreatment groups did not reveal statistically significant differences at T0–T1 and T0–T2 for cell counts (Supplementary Figures S8 and S9) and for most biochemical parameters (Supplementary Figures S10 and S11), indicating a similar course regardless of initial levels. The exception was LDH, where patients with high baseline values showed a significantly greater decrease at T2 compared with the low-value group (*p* < 0.01) (Figure 3).

In contrast, among cytokines, statistically significant differences at T1 were observed for fractalkine/CX3CL1, IL-2, IL-5, and MIP-1α/CCL3 (*p* < 0.05) and at T2 for ITAC/CXCL11, IL-4 (*p* < 0.05), and GM-CSF (*p* < 0.01) (Figure 3). Patients with low baseline levels of fractalkine/CX3CL1, IL-2, and MIP-1α/CCL3 demonstrated a sharp increase at T1, while patients with high baseline levels showed only a minimal decrease. Both ‘low’ and ‘high’ baseline groups showed increases in IL-5 at T1; however, the former was significantly increased compared to the latter. These motifs were not sustained at T2. On the contrary, some cytokines with non-significant fluctuations at T1 showed significant differences for T2. Namely, for ITAC/CXCL11 and GM-CSF, patients with low baseline levels exhibited a pronounced increase, whereas those with high baseline levels demonstrated minimal negative changes. For IL-4, patients with low baseline levels demonstrated a further decrease during the first three months of treatment, while those with high baseline levels showed minimal positive change. No significant changes were seen for the rest of cytokines for both T0–T1 and T0–T2 (Supplementary Figures S12 and S13).

Analysis for sPD-L1 showed significant early baseline kinetic changes for T0–T1 (*p* < 0.05), which were sustained at T2 (*p* < 0.05), with patients exhibiting low pretreatment levels presenting with pronounced increases at both timepoints compared to patients with high baseline levels (Figure 3).

#### 3.3.4. Assessment of Early Kinetics Stratified per Response Status

To evaluate whether treatment-induced changes differed according to clinical outcome, we assessed Δ% differences between ‘responders’ and ‘non-responders’ at early timepoints (T0–T1 and T0–T2). Comparisons of response-stratified kinetics at T3 and T4 were not pursued, as they were considered unlikely to provide meaningful insight into the predictive value of PBIMs and were further limited by patients’ attrition at later timepoints.

The Δ% comparisons of PBIMs per response status did not reveal any significant differences for T0–T1 (Supplementary Figures S14–S16) nor for T0–T2 (Supplementary Figures S17–S19).

#### 3.3.5. Changes Prior to Disease Progression

To assess significant alterations in the kinetics of parameters before PD, measurements immediately prior to progression (pre-PD) were compared with the preceding measurement values (reference values). Only patients with available samples collected within three months prior to PD were included in the analysis.

A total of 14 patients met the criteria and were used for the analysis. Comparative analysis did not reveal statistically significant changes for any of the examined parameters (Supplementary Figures S20–S22).

### 3.4. Association of Peripheral Blood Immune Markers with Therapeutic Outcomes

#### 3.4.1. Association with 6-Month Clinical Benefit (CB6)

For the evaluation of the prognostic value of PBIMs for CB6, univariate logistic regression was performed for the pretreatment values (T0) of all parameters and for the significant changes (Δ%) between T0–T1 and T0–T2 in the kinetic analysis. Detailed associations of all parameters with CB6 are summarized in Supplementary Table S5.

Assessment of cell counts and biochemical markers revealed a statistically significant negative association of baseline CRP (OR = 0.81, 95%CI: 0.63–0.98, *p* < 0.05) with CB6, as well as a negative trend for CAR (OR = 0.55, 95%CI: 0.24–1.02, *p* 0.06). A strong negative association was also observed for complement protein C4 baseline levels (OR = 0.85, 95%CI: 0.72–0.95, *p* < 0.01) and a positive correlation with complement ratio (OR = 4.43, 95%CI: 1.59–18.74, *p* < 0.01). Regarding cytokines, only baseline IL-6 levels demonstrated a statistically significant negative association (OR = 0.58, 95%CI: 0.31–0.92, *p* < 0.05). Baseline sPD-L1 levels did not show any significant association with CB6 (OR = 1.04, 95%CI: 0.87–1.30, *p* = 0.67).

The percentage changes (Δ%) of total leukocytes; neutrophils; CRP; LDH; sPD-L1; and cytokines, including ITAC/CXCL11, GM-CSF, fractalkine/CX3CL1, MIP-3α/CCL20, IL-17α, IL-1β, IL-2, IL-4, IL-5, IL-7, MIP-1α/CCL3, and TNFα, did not reveal significant associations with CB6.

#### 3.4.2. Association with Progression-Free Survival at 2 Years (2y PFS)

Univariate Cox regression analysis was performed to assess the prognostic association of PBIMs with 2y PFS, taking into account baseline values (T0) and statistically significant percentage changes (Δ%) up to T2. Results are summarized in Supplementary Table S6.

Among baseline values, higher CRP levels were significantly associated with an increased risk of PD (HR = 1.13, 95%CI: 1.01–1.24, *p* < 0.05), as was CAR (HR = 1.45, 95%CI: 0.99–20.20, *p* < 0.05), though the latter with wide and non-significant 95%CI. In addition, higher C4 levels were linked to increased PD risk (HR = 1.09, 95%CI: 1.03–1.50, *p* < 0.01), while a higher complement ratio showed a protective effect (HR = 0.52, 95%CI: 0.29–0.86, *p* < 0.01). No significant associations were identified for the remaining baseline values. Regarding cytokines, elevated baseline levels of IL-6 (HR = 1.63, 95%CI: 1.20–2.24, *p* < 0.01) and IL-10 (HR = 1.21, 95%CI: 1.03–1.40, *p* < 0.05) were significantly associated with worse outcomes, while a trend was observed for IL-12/p70 (HR = 1.33, 95%CI: 0.92–1.90, *p* = 0.09). No significant associations with 2y PFS were observed for the remaining cytokines. Baseline sPD-L1 was not significantly associated with 2y PFS (HR = 1.05, 95%CI: 0.94–1.16, *p* = 0.34).

The markers with significant early changes (Δ% between T0–T1 and T0–T2) in the longitudinal analysis were not significantly associated with 2y PFS.

#### 3.4.3. Stratification of Markers Prognostication According to Discriminative Ability

**Figure 4:**
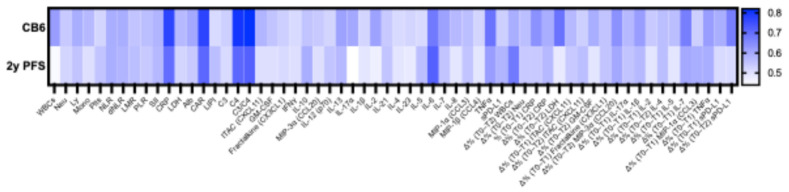
Heatmap of the discriminative ability of peripheral blood immune markers for the 6-month clinical benefit (expressed as AUC) and for the progression-free survival at 2 years (expressed as Harrel’s C-index). Abbreviations. Alb: albumin; AUC: area under the curve; CAR: CRP-to-albumin ratio; CB6: clinical benefit at 6 months; CCL3, 4, 20: C-C motif chemokine ligand; CI: confidence interval; CRP: C-reactive protein; CXCL11: C-X-C motif chemokine ligand 11; CX3CL1: C-X3-C motif chemokine ligand 1; C3/4: complement proteins; C3/C4: C3-to-C4 ratio; dNLR: derived NLR; IFN-γ: interferon-γ; IL: interleukin; ITAC: interferon-inducible T-cell alpha chemoattractant; LDH: lactate dehydrogenase; LIPI: Lung Immune Prognostic Index; LMR: lymphocyte-to-monocyte ratio; Ly: lymphocytes; MIP-3α: macrophage inflammatory protein α; Mono: monocytes; Neu: neutrophils; NLR: neutrophil-to-lymphocyte ratio; PLR: platelet-to-lymphocyte ratio; Plts: platelets; SII: Systemic Inflammation Index; sPD-L1: soluble programmed-death ligand-1; T0: pretreatment (baseline); T1: 21 days (before cycle 2); T2: 3 months; WBCs: white blood cells; Δ%: percentage change; 2y PFS: progression-free survival at 2 years.

The PBIMs assessed with univariate logistic regression for their association with the CB6 were further evaluated for their discriminative performance using AUC (calculated from the ROC curve). Discriminate ability of PBIMs for 2y PFS was assessed using Harrel’s C-index. The AUC/C-index values for all parameters, in relation to clinical outcomes, are illustrated in Figure 4.

Among the PBIMs with acceptable discriminative ability, there was a significant negative association with the baseline levels of CRP (AUC = 0.77, 95%CI: 0.60–0.94, OR = 0.81, 95%CI: 0.63–0.98, *p* < 0.05) and C4 protein (AUC = 0.80, 95%CI: 0.64–0.96, OR = 0.85, 95%CI: 0.72–0.95, *p* < 0.01) with CB6, and a significant positive association with C3/C4 ratio (AUC = 0.82, 95%CI: 0.67–0.97, OR = 4.43, 95%CI: 1.59–18.74, *p* < 0.01). There was also a non-significant negative trend of CB6 with CAR (AUC = 0.77, 95%CI: 0.59–0.95, OR = 0.55, 95%CI: 0.24–1.02, *p* = 0.06) (Table 2).

For 2y PFS, among PBIMs with acceptable discriminative ability, baseline CRP (C-index = 0.70, 95%CI: 0.58–0.79, HR = 1.13, 95%CI: 1.01–1.24, *p* < 0.05), C4 (C-index = 0.71, 95%CI: 0.59–0.83, HR = 1.09, 95%CI: 1.03–1.15, *p* < 0.01), and IL-6 (C-index = 0.72, 95%CI: 0.54–0.81, HR = 1.63, 95%CI: 1.20–2.24, *p* < 0.01) values showed a significant correlation with worse outcomes. The complement ratio showed a significant negative correlation with 2y PFS (C-index = 0.72, 95%CI: 0.60–0.83, HR = 0.52, 95%CI: 0.29–0.86, *p* < 0.05) (Table 2).

#### 3.4.4. Multivariable Marker Analysis and Composite Prognostic Scores

After the initial univariate analysis and the selection of parameters that demonstrated acceptable discriminative ability and significant association with clinical endpoints, further exploratory multivariable analysis with adjustment to established clinical prognostic factors was sought. ECOG PS, presence of brain metastases, and number of organs involved were included in multivariable models as independent prognostic factors for NSCLC patients treated with ICIs have been consistently reported in the literature [25,26,27,28]. ECOG PS and brain metastases were used as a binary variable (good PS = 0–1 vs. worse PS ≥ 2 and presence of brain metastases vs. no brain metastases), while ‘distant metastases’ was included as a continuous variable expressing the number of organs involved.

Baseline values of CRP and C4 were selected for further evaluation in the multivariate adjustment for their association with CB6 and 2y PFS. Baseline values of IL-6 were also included in the adjusted analysis of 2y PFS. Despite the significant association of complement ratio with both clinical outcomes, it was not included in the multivariate analysis due to the wide 95%CIs (high-to-low CI ≥ 3), suggesting instability of the estimate, while one of its individual components (complement C4) was already incorporated. Adjusted ORs/HRs for PBIMs are reported in Table 3.

For CB6, both CRP and C4 retained their negative prognostic value; however, the former lost its statistical significance (OR = 0.80, 95%CI: 0.52–1.13, *p* = 0.22) while the latter was significantly associated with negative CB6 outcomes (OR = 0.84, 95%CI: 0.68–0.96, *p* < 0.01). Moreover, their integration into a composite score (including both CRP and C4) further strengthened the predictive power in an independent multivariable model, showing a strong association with reduced CB6 (OR = 0.23, 95%CI: 0.06–0.60, *p* < 0.01) (Table 3).

For the 2y PFS, CRP (HR = 1.01, 95%CI: 0.89–1.14, *p* = 0.84) and IL-6 (HR = 1.37, 95%CI: 0.93–2.00, *p* = 0.11) lost their statistical significance in the multivariable analysis; however, C4 retained its negative prognostic impact (HR = 1.10, 95%CI: 1.03–1.20, *p* < 0.01). The combined index of these three parameters (CRP, C4, IL-6) demonstrated a significantly strong negative prognostic value (HR = 2.40, 95%CI: 1.45–4.28, *p* < 0.001) (Table 3).

#### 3.4.5. Sensitivity Analyses for Potential Clinical Confounders

Sensitivity analyses were performed to assess whether the prognostic associations of baseline CRP, C4, and IL-6 were influenced by baseline corticosteroid/immunomodulatory treatment or recent radiotherapy exposure. Considering the cohort size, a sensitivity analysis excluding both patients who had received radiotherapy (n = 18, 58.0%) and corticosteroids or immunomodulators (n = 14, 45.2%) was not feasible, as the remaining non-exposed subgroup (n = 9, 29.0%) would render any longitudinal or multivariable statistics unreliable. Consequently, sensitivity analyses were restricted to separate univariate models according to exposure status for each potential confounder.

Among patients without recent radiotherapy exposure, baseline CRP and C4 retained negative associations with CB6. C4 remained statistically significant (AUC = 0.88, 95%CI: 0.68–1.00; OR = 0.77, 95%CI: 0.54–0.94; *p* < 0.01), while CRP also maintained a significant association (AUC = 0.80, 95%CI: 0.49–1.00; OR = 0.65, 95%CI: 0.34–0.98; *p* = 0.04). Similarly, among patients not receiving corticosteroids or other immunomodulatory agents at baseline, C4 maintained a significant negative association with CB6 (AUC = 0.92, 95%CI: 0.76–1.00; OR = 0.77, 95%CI: 0.55–0.92; *p* < 0.01), whereas CRP demonstrated a non-significant trend (AUC = 0.83, 95%CI: 0.64–1.00; OR = 0.81, 95%CI: 0.58–1.04; *p* = 0.10).

For 2y PFS, the associations of CRP, C4, and IL-6 with progression risk were attenuated in both sensitivity analyses and did not retain statistical significance. In patients without recent radiotherapy exposure, CRP (C-index = 0.45, 95%CI: −0.12–1.03; HR = 1.27, 95%CI: 0.91–1.89; *p* = 0.13), C4 (C-index = 0.66, 95%CI: 0.22–1.09; HR = 1.05, 95%CI: 0.89–1.22; *p* = 0.46), and IL-6 (C-index = 0.64, 95%CI: 0.18–1.09; HR = 1.95, 95%CI: 0.77–6.28; *p* = 0.15) showed directionally consistent but statistically non-significant associations with worse outcomes. Similarly, in patients not receiving corticosteroids or immunomodulatory agents, CRP (C-index = 0.67, 95%CI: 0.28–1.05; HR = 1.05, 95%CI: 0.91–1.18; *p* = 0.46), C4 (C-index = 0.74, 95%CI: 0.51–0.97; HR = 1.03, 95%CI: 0.97–1.09; *p* = 0.38), and IL-6 (C-index = 0.46, 95%CI: 0.03–0.88; HR = 1.10, 95%CI: 0.66–1.80; *p* = 0.70) did not retain statistical significance. The attenuation of significance likely reflects the substantial reduction in sample size and event numbers within these exploratory subgroup analyses. Detailed results are summarized in Supplementary Table S7.

## 4. Discussion

In this prospective study, we implemented longitudinal PBIMs monitoring in a treatment-naïve, PD-L1 high NSCLC cohort treated with first-line pembrolizumab monotherapy. Serial sampling across the first treatment year enabled characterization of PBIMs trajectories and an exploratory assessment of baseline levels and early in-treatment kinetics in relation to early (CB6) and sustained (2y PFS) benefit after two years of follow-up was performed.

Across the first year of treatment, inflammatory burden decreased, most consistently reflected by a sustained decline in CRP and accompanied by reductions in leukocyte/neutrophils and MIP-3α/CCL20 at three months. IL-17α, IL-6, and IL-8 also declined over time consistent with attenuation of pro-inflammatory signaling during treatment. At three weeks, transient increases in selected cytokines (ITAC/CXCL11, IL-1β, IL-7, TNFα) were observed and may represent an early immune activation (“flare”) following PD-1 blockade, as has been previously reported [28,29], preceding later immune adaptation.

The role of CRP in cancer has been previously studied and linked with worse outcomes in NSCLC [11]. The decline of CRP during treatment in our study may be reflective of de-escalation of tumor-associated inflammation. The latter is also supported by the marked leukocyte/neutrophils decrease during early treatment. The cytokines IL-6 and IL-8 have been repeatedly associated with poor prognosis and immunotherapy resistance [15]. These cytokines standout in the pro-inflammatory cascade of several inflammatory conditions, including cancer, and are mediators of carcinogenesis via the STAT3 pathway [30,31,32,33]. IL-17α has also been associated with tumor progression and immunotherapy resistance by increasing PD-L1 expression, inhibiting autophagy, and promoting neutrophil infiltration into the TIME [34,35]. Moreover, the role of chemokine MIP-3α/CCL20 in tumor growth and in the promotion of an immunosuppressive environment has been previously described [36,37]. While these markers would be expected to decrease as a result of the immune adaptation after the introduction and the subsequent disease control of ICIs, their declining levels may also be confounded by the dropout of patients with high inflammatory burden at baseline and subsequent worse clinical outcomes.

Exploratory stratification by baseline ‘low’ vs. ‘high’ levels aimed to examine immune adaptation among patients with values outside the mean baseline distribution. Although median dichotomization does not fully capture biological phenotypes, distinct dynamic patterns emerged. Several cytokines demonstrated a pronounced early increase in the ‘low’ baseline subgroup at T1 (fractalkine/CX3CL1, IL-2, IL-5, MIP-1α/CCL3), consistent with an acute immune activation following PD-1 blockade, while others increase at T2 (ITAC/CXCL11, GM-CSF), suggesting subsequent immune adaptation. Conversely, patients with high baseline LDH levels showed a marked decline at T2, in line with decreasing tumor burden during treatment, as previously reported [38]. A sharp rise in sPD-L1 was observed in the ‘low’ baseline subgroup at both T1 and T2. Given the inconsistent literature on sPD-L1 kinetics we hypothesize that this early increase may reflect reduced ligand availability on tumor cells following effective PD-1 inhibition [16,17,39,40]. The pronounced dynamic changes primarily observed in the ‘low’ baseline subgroups may reflect immune engagement in patients with initially suppressed or quiescent immune signaling, whereas the relative stability of the ‘high’ baseline groups suggests a ‘ceiling’ effect or a pre-existing inflamed state with limited capacity for activation following PD-1 blockade. Despite these interesting findings, this analysis should be interpreted with caution, as median dichotomization may not fully capture the behavior of continuously distributed markers, as illustrated by the significant decrease in IL-4 ‘low’ baseline subgroup at T2, where the magnitude of Δ% appeared small and not reflective of the enhanced results in the statistical analysis. Moreover, an important methodological consideration in the Δ% analyses is the sensitivity of numerically small changes to reflect large relative (%) changes. This was particularly pronounced in patients with baseline values close to the LOD. While the observed large percentage changes may partly reflect mathematical amplification due to low baseline values, it cannot be excluded that they also represent true biological effects, whereby patients with low or near-baseline levels exhibit pronounced relative changes following treatment. However, given the potential instability of these estimates, such findings should be interpreted with caution and considered exploratory. An alternative analysis of patients at the baseline extremes was not pursued due to the sample constraints and potential statistical pitfalls.

Furthermore, PBIMs kinetics were examined according to response status and in the pre-progression (pre-PD) period for patients who subsequently developed disease progression. The response-stratified analysis aimed to investigate the potential predictive role of PBIMs dynamics, while the pre-PD analysis sought to explore fluctuations that might signal impending progression prior to radiological assessment or in cases of ambiguous restaging findings (e.g., tumor increase classified as SD per RECIST or ambiguous new lesions). However, no significant associations were observed in either analysis, potentially due to the limited number of available measurements.

Next, a statistical filtering approach was applied to identify prognostic PBIMs for each clinical endpoint. From a statistical point of view, given the exploratory nature of this study and the relatively small sample size, no formal adjustments for multiple comparisons (e.g., Bonferroni correction) was applied, as such methods may be overly conservative and increase the risk of type II error in hypothesis-generating analyses. Instead, a multi-step filtering strategy was implemented to mitigate false-positive findings, including assessment of discriminative performance (AUC/C-index thresholds), evaluation of confidence intervals, and confirmation in multivariable models. These approaches were intended to prioritize biologically and clinically meaningful associations over isolated statistically significant findings. Therefore, all results should be interpreted as exploratory and hypothesis-generating, requiring validation in independent cohorts.

Among markers with sufficient discriminatory ability, only baseline complement protein C4 retained a significant negative prognostic association with both CB6 and 2y PFS in multivariable analysis. Notably, integrating C4 with other significant univariate markers (baseline CRP for CB6 and 2y PFS, and IL-6 for 2y PFS) into composite indices further improved prognostic performance, highlighting the value of immune profile integration. These markers may capture complementary and potentially independent aspects of the host immune and inflammatory status prior to immunotherapy introduction. While the adverse prognostic roles of CRP, and IL-6 are well established [11,15,41], the prognostic significance of C4 in the immunotherapy setting has not been previously reported.

Although prior data associated lower C3– but not C4– with improved outcomes [14], our findings suggest that C4 may act as a negative biomarker, consistent with its role in promoting an immunosuppressive TIME [42]. Mechanistically, complement activation is increasingly recognized as driver of tumor immune evasion. While C4 is a recognized acute phase reactant in response to inflammatory conditions, such as infections and autoimmune diseases, in cancer, as an upstream component of the classical and lectin pathways, higher circulating C4 may reflect heightened baseline complement tone that can propagate downstream activation, including generation of immunomodulatory fragments (e.g., C3a/C5a) known to shape a suppressive TIME [42]. However, elevated detection of C4 in our study, and association with poor outcomes, may reflect the circulating levels due to systemic inflammation rather than the immunosuppression exerted in the TIME. Complement signaling has been shown to promote recruitment and function of suppressive myeloid populations, dampen effective T-cell responses, and support tumor-associated inflammatory processes that could antagonize PD-1 antitumor immunity [43]. In preclinical lung cancer models, blockade of the C5a axis has enhanced the efficacy of PD-1 blockade, supporting a functional link between complement activity and ICI responsiveness [44]. Recent work has also highlighted that C4 activation products (e.g., C4d) are detectable in tumors and may represent a component of complement biology in cancer [42]. Together, these observations provide biological plausibility for our finding that elevated baseline C4 associates with poorer outcomes; nevertheless, these findings should be interpreted with caution as the available literature is limited and not fully consistent, with most prior reports focusing on C3 rather than C4, and the biological role of individual complement components in the context of ICIs remains incompletely understood. Further studies in larger, independent cohorts are required to clarify the contribution of different complement components in immunotherapy.

Importantly, in the present cohort, meaningful prognostic associations were primarily observed for baseline biomarker levels, whereas longitudinal analyses, including response-stratified kinetics and pre-progression fluctuations, did not demonstrate significant associations with clinical outcomes. While the limited number of available measurements and patient attrition may have contributed to these negative findings, this pattern may also reflect a potency of baseline immune and inflammatory status in determining immunotherapy outcomes compared to short- and long-term dynamic changes during treatment. Therefore, while longitudinal PBIM monitoring provided valuable descriptive insights into immune dynamics, its prognostic utility appeared limited in this cohort compared with baseline biomarker assessment.

In addition, in the present study, several blood-based indices that have previously been predictive for immunotherapy outcomes, including LIPI score, SII, NLR, dNLR, PLR, albumin-related indices, and others, were assessed (Supplementary Tables S4 and S5); however, these indices did not demonstrate significant associations with clinical outcomes in our cohort. This discrepancy may be explained by several factors. First, our study population was restricted to patients with PD-L1 high disease representing a biologically selected and relatively homogenous cohort with a known favorable factor for immunotherapy outcomes. In this setting, generalized inflammatory indices may be less sensitive than more specific immune and inflammatory mediators. In contrast, baseline CRP, C4, and IL-6 demonstrated association with outcomes, suggesting that more granular blood-based immune markers may provide additional prognostic information in this selected population. Second, the limited sample size and event number reduce statistical power, particularly for categorical or composite scores, such as LIPI and SII. These findings remain hypothesis-generating and require validation in larger cohorts.

Lastly, this prospective study carries several strengths and limitations. It provides a comprehensive longitudinal evaluation of PBIM kinetics in a chemotherapy-naïve PD-L1 high NSCLC cohort that was treated uniformly with pembrolizumab monotherapy, enabling characterization of immune dynamics across early and later timepoints. The broad biomarker panel and extended follow-up identified inflammatory trajectories and early immune activation effects, explored prognostic relevance of previously unreported markers (complement C4), and highlighted the significance of composite PBIMs profiles. However, the single-center design, limited sample size, and patient attrition at later timepoints restrict statistical power and may confound exploratory outcome associations. Importantly, the relatively small cohort size limits the statistical power of subgroup analyses and multivariable models, increasing the risk of overfitting and wide confidence intervals. Therefore, the observed associations should be considered hypothesis-generating rather than definitive. Furthermore, the selection of CB6 as an early endpoint for treatment benefit combined CR, PR, and SD into one group, which may create a broad and biologically heterogenous endpoint. In addition, patient attrition over time, mainly due to disease progression, may have introduced survivorship bias in longitudinal analyses, especially at later timepoints. This may partially explain the attenuation of certain dynamic signals over time. To partially address this limitation, baseline characteristics were compared between patients with complete and incomplete longitudinal sampling, and no statistically significant imbalances were identified; however, the limited cohort size restricts the ability of such analyses to fully exclude survivorship bias. Furthermore, the absence of an internal validation or an independent external validation cohort limits the generalizability of the findings. External validation in larger, multicenter prospective studies will be essential to confirm the reproducibility and clinical applicability of these results. Lastly, a substantial proportion of patients received baseline radiotherapy and/or corticosteroids or other immunomodulatory agents, which are known to influence systemic immune and inflammatory markers. Radiotherapy may induce cytokine release and immune modulation, while corticosteroids can suppress immune activation and alter circulating cytokine profiles and immune cells recruitment, potentially confounding both PBIM kinetics and their associations with clinical outcomes. Due to the cohort size, a combined exclusion sensitivity analysis for both exposures was not feasible, as the remaining non-exposed subgroup (n = 9, 29.0%) would render longitudinal and multivariable analyses unreliable. Therefore, exploratory sensitivity analyses were restricted to separate univariate subgroup analyses according to radiotherapy and corticosteroid/immunomodulatory exposure status. In these analyses, the negative association of baseline C4 with CB6 remained statistically significant across both subgroups, while the associations of CRP, C4, and IL-6 with 2y PFS generally retained directional consistency but lost statistical significance. However, given the markedly reduced subgroup sizes, these analyses were substantially underpowered and associated with unstable estimates and wide confidence intervals. Consequently, they should not be considered reliable for definitive statistical inference, but rather as exploratory assessments of the robustness and directionality of the primary findings. Validation in larger prospective cohorts is therefore warranted.

From a clinical perspective, PBIMs offer a minimally invasive and repeatable approach that could complement existing tissue-based biomarkers. If validated, such markers could be incorporated into routine monitoring to provide early indications of treatment response or resistance, potentially guiding treatment decisions such as continuation, intensification, or modification of therapy. However, standardized thresholds, validation across populations, and integration with clinical and radiological parameters will be required before clinical implementation.

## 5. Conclusions

This prospective study provides descriptive longitudinal data on PBIM dynamics in PD-L1 high NSCLC patients treated with anti-PD-1 monotherapy and offers exploratory insight into their potential prognostic relevance. We observed a consistent decline in key inflammatory mediators, suggesting attenuation of systemic inflammation during treatment, along with heterogeneous adaptive immune responses across baseline subgroups. In exploratory analyses, baseline CRP, IL-6, and especially complement C4 were associated with unfavorable clinical outcomes, and integrated PBIM composite profiles showed enhanced discriminatory performance. While these findings remain exploratory and are limited by the small single-center cohort, they support the feasibility of longitudinal PBIM monitoring as a minimally invasive approach to characterize tumor–immune interactions and warrant validation in larger prospective studies.

## Figures and Tables

**Table 1:** Summary of collected samples and treatment infusions at the pre-specified timepoints.

Pre-Specified Time Points	T0	T1	T2	T3	T4
Number of samples	31	30	27	21	20
Number of pembrolizumab infusions *	-	1	4 (2–4)	7 (5–8)	13 (8–15)

T0: Pretreatment (baseline), T1: 21 days, T2: 3 months, T3: 6 months, T4: 1 year; * Median (IQR).

**Table 2:** Univariate analysis for the association of the peripheral blood immune markers with acceptable discriminative (AUC/C-index > 0.7, lower 95%CI > 0.5) with 6-month clinical benefit and progression-free survival at 2 years.

6-Month Clinical Benefit					
**Parameter**	**AUC**	**95%CI**	**OR**	**95%CI**	***p*** **-Value**
CRP	0.77	0.60–0.94	0.81	0.63–0.98	0.02
CAR	0.77	0.59–0.95	0.55	0.24–1.02	0.06
C4	0.80	0.64–0.96	0.85	0.72–0.95	<0.01
Complement ratio (C3/C4)	0.82	0.67–0.97	4.43	1.59–18.74	<0.01
**Progression-Free Survival at 2 Years**					
**Parameter**	**C-index**	**95%CI**	**HR**	**95%CI**	***p*** **-Value**
CRP	0.70	0.58–0.79	1.13	1.01–1.24	0.01
C4	0.71	0.59–0.83	1.09	1.03–1.15	<0.01
Complement ratio (C3/C4)	0.72	0.60–0.83	0.52	0.29–0.86	0.02
IL-6	0.72	0.54–0.81	1.63	1.20–2.24	<0.01

AUC: area under the curve; CAR: CRP-to-albumin ratio; CI: confidence interval; CRP: C-reactive protein; C3 and 4: complements protein 3 and 4; HR: hazard ratio; IL: interleukin; OR: odds ratio.

**Table 3:** Multivariate analysis for the adjustment of significant peripheral blood immune prognosticators from the univariate analysis to established clinical prognostic factors.

6-Month Clinical Benefit			
**Parameter**	**Adjusted OR**	**95%CI**	***p*** **-Value**
ECOG PS poor (≥2)	1.12	0.05–25.54	0.94
Brain metastases	0.30	0.01–5.24	0.42
Distant metastases (number of organs involved)	1.59	0.27–14.00	0.62
CRP	0.80	0.52–1.13	0.22
C4	0.84	0.68–0.96	<0.01
ECOG PS poor (≥2)	0.24	0.02–2.57	0.25
Brain metastases	0.26	0.02–3.41	0.31
Distant metastases (number of organs involved)	0.97	0.23–4.45	0.97
Composite score (CRP, C4)	0.23	0.06–0.60	<0.01
**Progression-Free Survival at 2 Years**			
**Parameter**	**Adjusted HR**	**95%CI**	***p*** **-Value**
ECOG PS poor (≥2)	0.51	0.10–2.55	0.42
Brain metastases	1.65	0.32–11.47	0.56
Distant metastases (number of organs involved)	0.96	0.38–2.24	0.94
CRP	1.01	0.89–1.14	0.84
C4	1.10	1.03–1.20	<0.01
IL-6	1.37	0.93–2.00	0.11
ECOG PS poor (≥2)	1.08	0.34–3.54	0.89
Brain metastases	1.41	0.36–6.87	0.64
Distant metastases (number of organs involved)	1.09	0.39–2.75	0.87
Composite score (CRP, C4, IL-6)	2.40	1.45–4.28	<0.001

CI: confidence interval; CRP: C-reactive protein; C4: complement protein 4; ECOG PS: Eastern Cooperative Oncology Group performance status; HR: hazard ratio; IL: interleukin; OR: odds ratio.

## Data Availability

Raw data are included in the Supplementary Material. Statistical analysis (.prism documents) are available upon request to the corresponding author (via email to itrontzas@med.uoa.gr).

## Supplementary Materials

The following supporting information can be downloaded at: https://www.mdpi.com/article/10.3390/cancers18101623/s1, Figure S1: Schematic illustration of clinical follow-up schedule and prespecified timepoints for peripheral blood immune markers assessment; Figure S2: Blood counts with non-significant fluctuations during treatment; Figure S3: Biochemical parameters with non-significant fluctuations during treatment; Figure S4: Cytokines and soluble PD-L1 without significant fluctuations during treatment; Figure S5: Blood counts with non-significant early changes (T0–T1 and T0–T2 kinetics); Figure S6: Biochemical parameters with non-significant early changes (T0–T1 and T0–T2 kinetics); Figure S7: Cytokines and soluble PD-L1 without significant early changes (T0–T1 and T0–T2 kinetics); Figure S8: Comparison of early dynamics (Δ%, T0–T1) between ‘low’ and ‘high’ baseline values of blood counts; Figure S9: Comparison of early dynamics (Δ%, T0–T2) between ‘low’ and ‘high’ baseline values of blood counts; Figure S10: Comparison of early dynamics (Δ%, T0–T1) between ‘low’ and ‘high’ baseline values of biochemical parameters; Figure S11: Comparison of early dynamics (Δ%, T0–T2) between ‘low’ and ‘high’ baseline values of biochemical parameters; Figure S12: Comparison of early dynamics (Δ%, T0–T1) between ‘low’ and ‘high’ baseline values of cytokines; Figure S13: Comparison of early dynamics (Δ%, T0–T2) between ‘low’ and ‘high’ baseline values of cytokines; Figure S14: Comparison of blood counts kinetics (Δ%) for T0–T1 between ‘responders’ and ‘non-responders’; Figure S15: Comparison of biochemical parameters kinetics (Δ%) for T0–T1 between ‘responders’ and ‘non-responders’; Figure S16: Comparison of cytokines and sPD-L1 kinetics (Δ%) for T0–T1 between ‘responders’ and ‘non-responders’; Figure S17: Comparison of blood counts kinetics (Δ%) for T0–T2 between ‘responders’ and ‘non-responders’; Figure S18: Comparison of biochemical parameters kinetics (Δ%) for T0–T2 between ‘responders’ and ‘non-responders’; Figure S19: Comparison of cytokines and sPD-L1 kinetics (Δ%) for T0–T2 between ‘responders’ and ‘non-responders’; Figure S20: Assessment of pre-progression dynamics of blood counts; Figure S21: Assessment of pre-progression dynamics for biochemical parameters; Figure S22: Assessment of pre-progression dynamics for cytokines and soluble PD-L1; Table S1: Summary of the cytokines/chemokines analyzed with the MILLIPLEX^®^ MAP Human High Sensitivity T Cell Panel and their lower limits of detection (LOD); Table S2: Baseline clinical and pathological characteristics; Table S3: Response and survival outcomes at the 2-year follow-up; Table S4: Comparison of baseline clinicopathological characteristics between patients who completed (completers) the 1-year follow-up and those who did not (non-completers); Table S5: Association between the pretreatment/baseline (T0) values and the significant early changes (Δ%, T0–T1 and Τ0–Τ2) of the peripheral blood immune markers with 6-month clinical benefit (CB6), assessed with univariate logistic regression; Table S6: Association between the pretreatment/baseline (T0) values and the significant early changes (Δ%, T0–T1 and Τ0–Τ2) of the peripheral blood immune markers with progression-free survival at 2 years (2y PFS), assessed with univariate Cox regression; Table S7: Sensitivity analyses for potential clinical confounders (recent radiotherapy exposure and baseline corticosteroid/immunomodulatory treatment) of significant PBIMs from multivariable prognostic analyses. Univariate models were used for association of baseline levels of PBIMs between patients who had not received radiotherapy or corticosteroids/immunomodulators and clinical outcomes; Raw Data Supplement.
