# Supplementary material for: In-Treatment Kinetics of Peripheral Blood Immune Markers in PD-L1 High Non-Small Cell Lung Cancer and Prognostic Relevance for Immunotherapy Outcomes
Source: Cancers (Basel). 2026 May 17;18(10):1623. doi: 10.3390/cancers18101623 (PMC13204403; doi:10.3390/cancers18101623)
Supplement: Supplementary file 1 [file cancers-18-01623-s001.zip › Figures S1-22.pptx]

## Slide 1
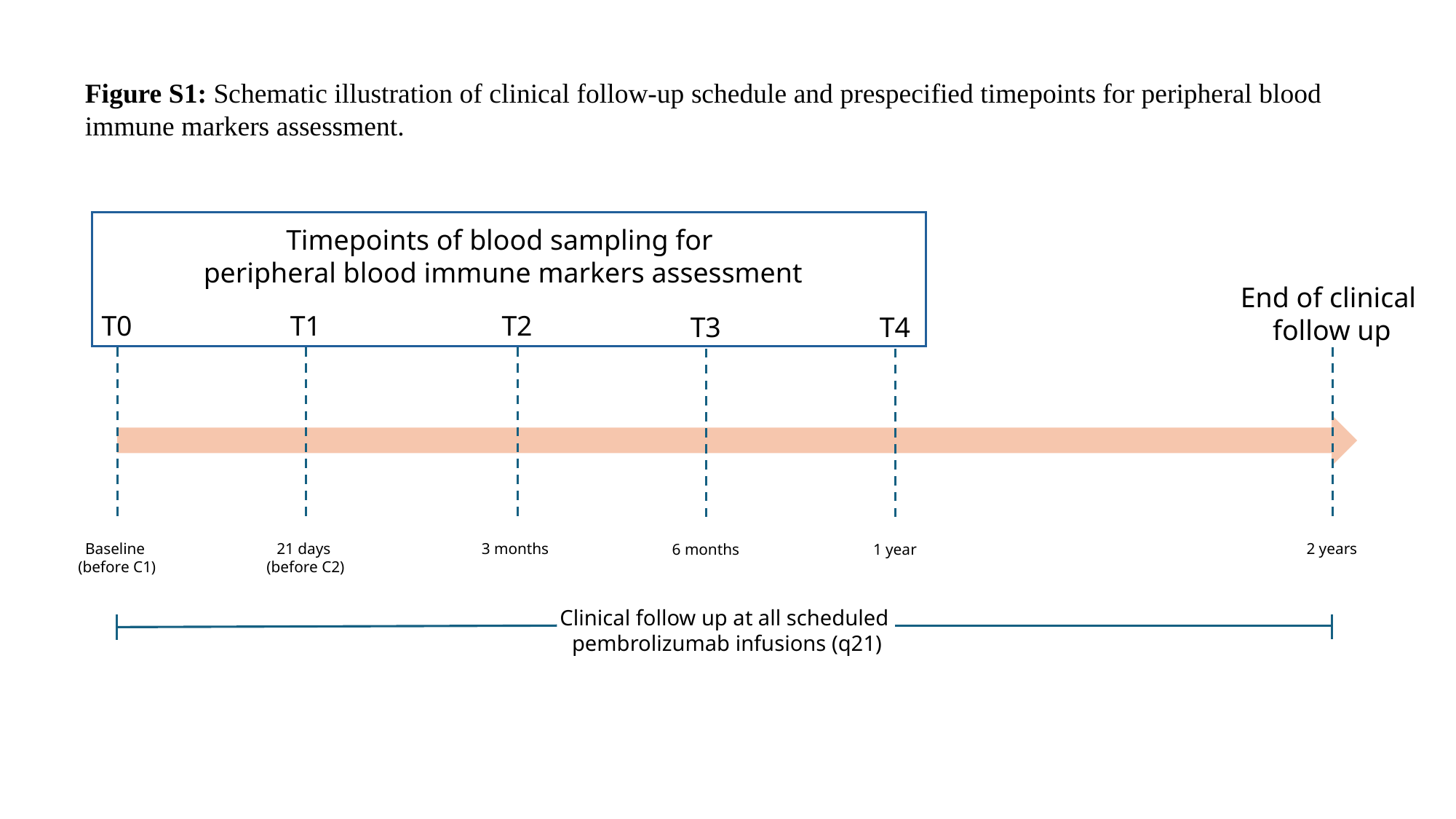

Figure S1: Schematic illustration of clinical follow-up schedule and prespecified timepoints for peripheral blood
immune markers assessment.
Timepoints of blood sampling for
peripheral blood immune markers assessment
End of clinical
follow up
T0
T1
T2
T3
T4
Baseline
(before C1)
21 days
(before C2)
3 months
2 years
6 months
1 year
Clinical follow up at all scheduled
pembrolizumab infusions (q21)

## Slide 2
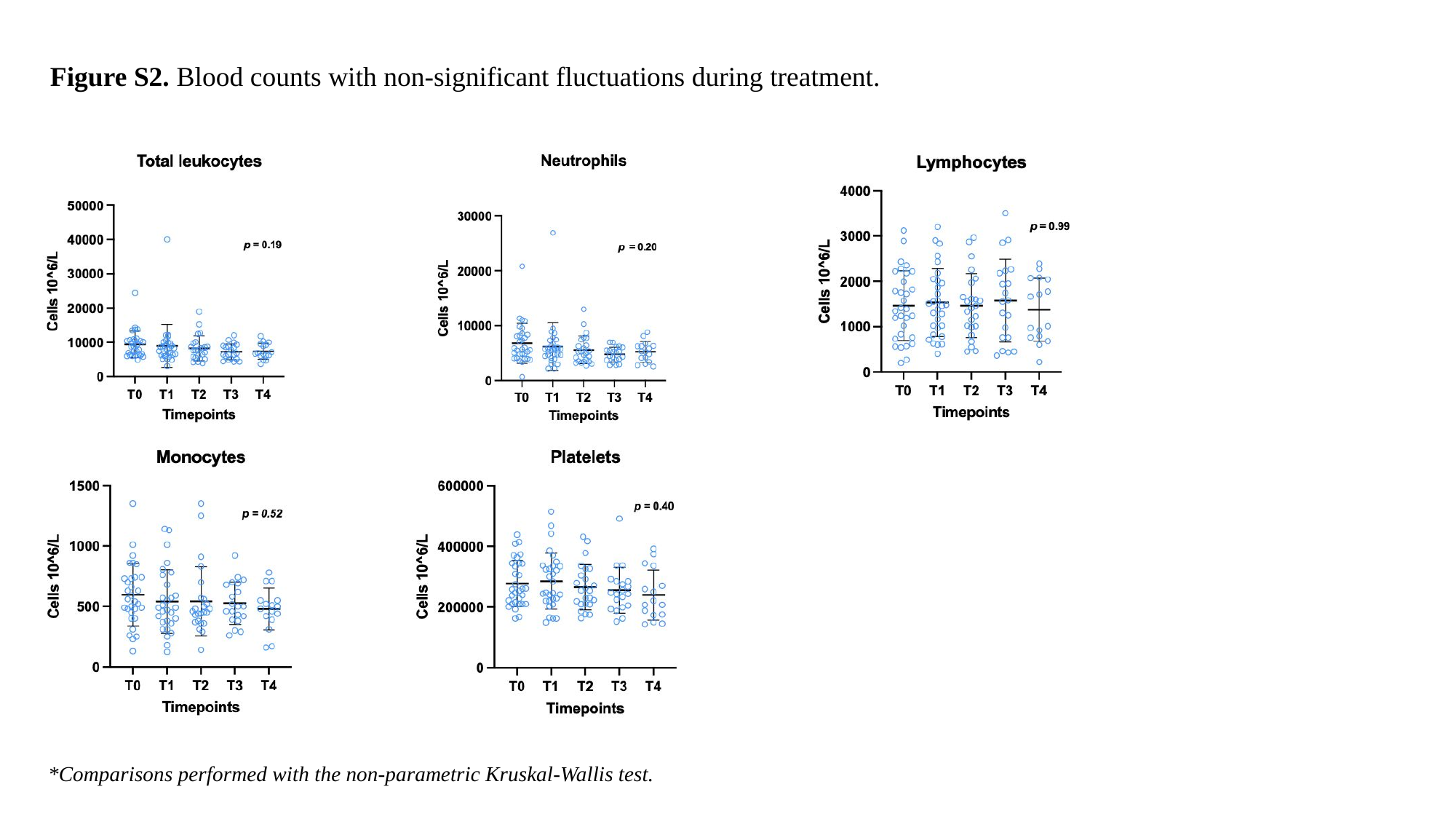

Figure S2. Blood counts with non-significant fluctuations during treatment.
*Comparisons performed with the non-parametric Kruskal-Wallis test.

## Slide 3
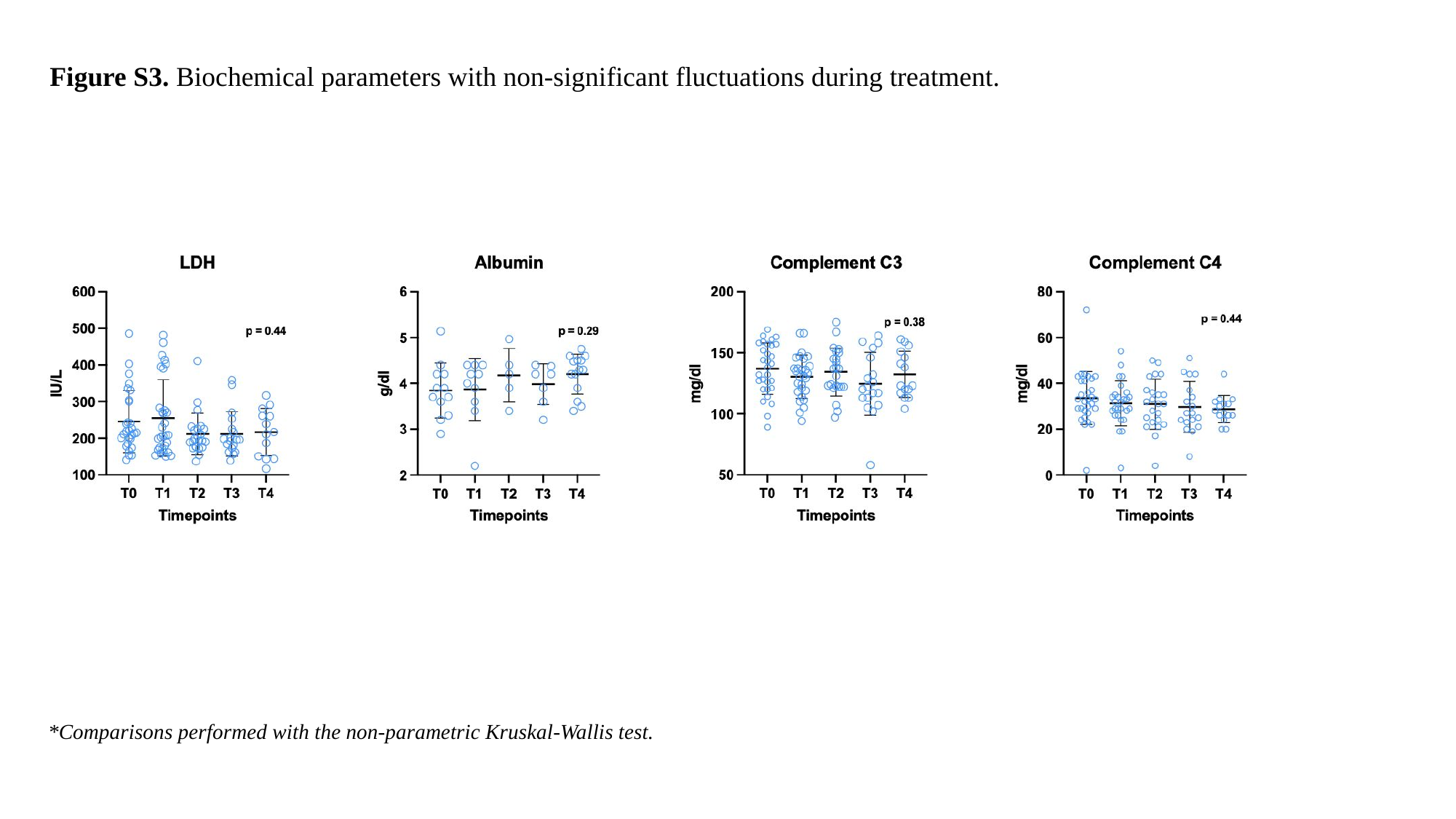

Figure S3. Biochemical parameters with non-significant fluctuations during treatment.
*Comparisons performed with the non-parametric Kruskal-Wallis test.

## Slide 4
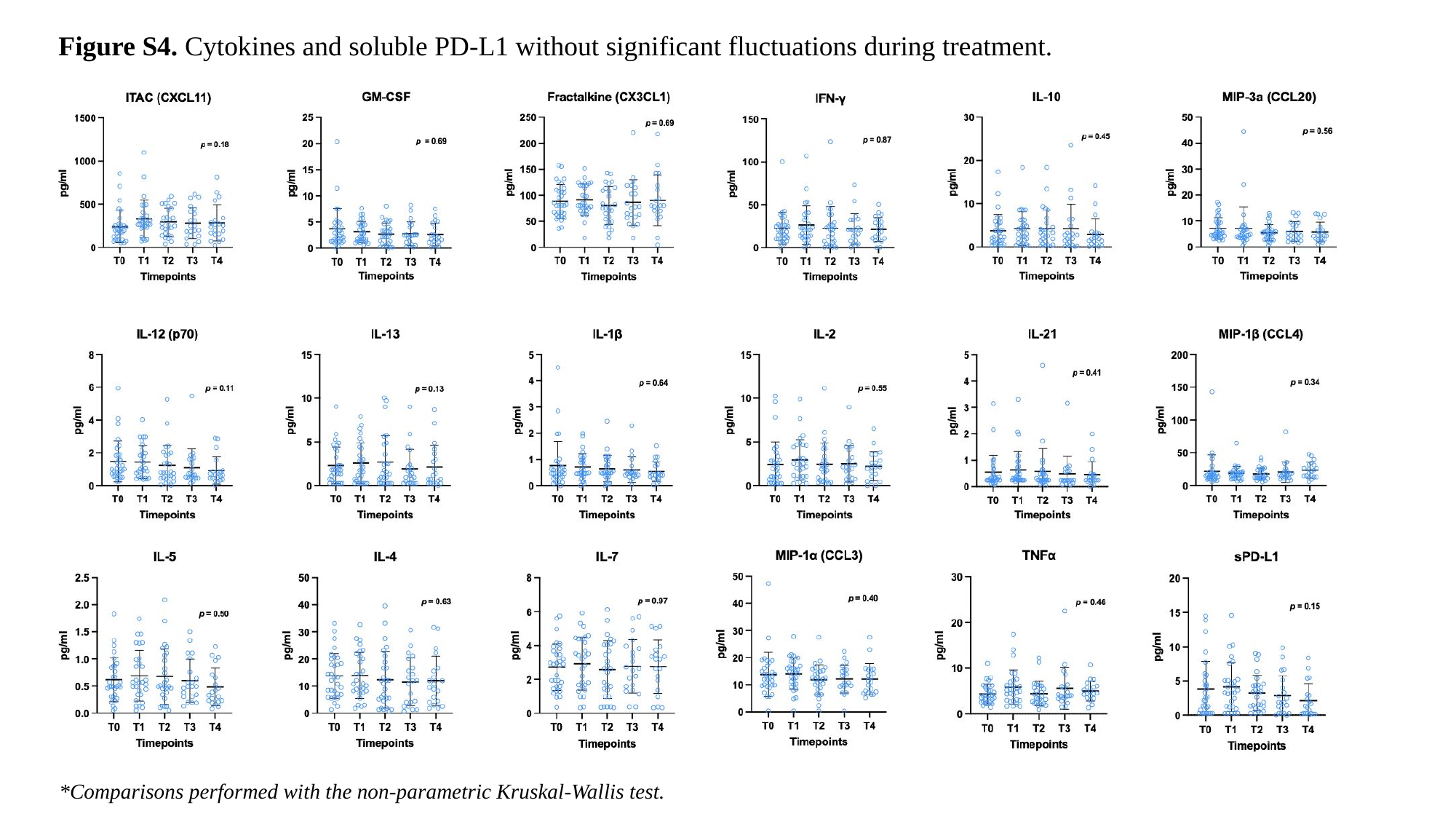

Figure S4. Cytokines and soluble PD-L1 without significant fluctuations during treatment.
*Comparisons performed with the non-parametric Kruskal-Wallis test.

## Slide 5
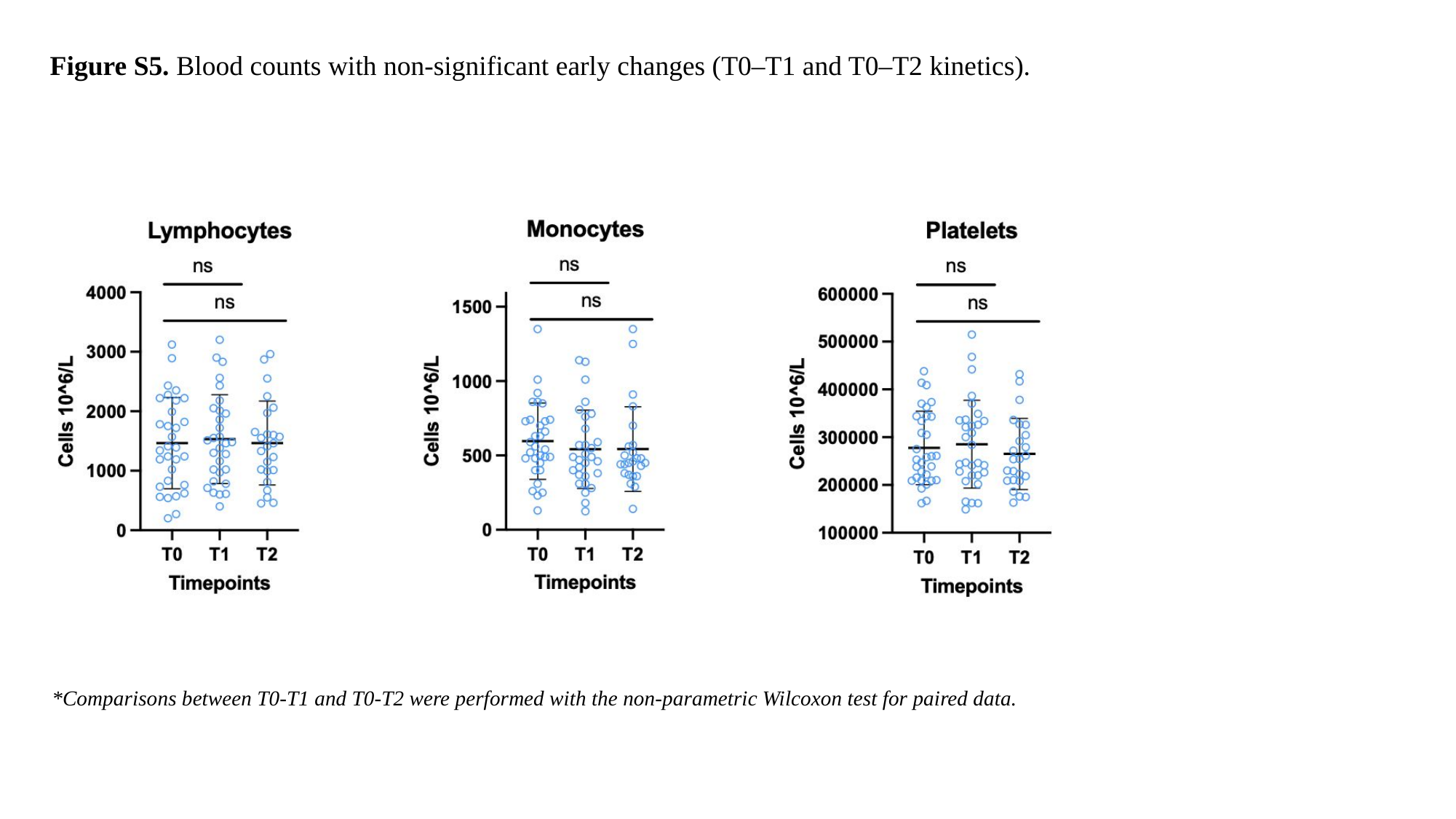

Figure S5. Blood counts with non-significant early changes (T0–T1 and T0–T2 kinetics).
*Comparisons between T0-T1 and T0-T2 were performed with the non-parametric Wilcoxon test for paired data.

## Slide 6
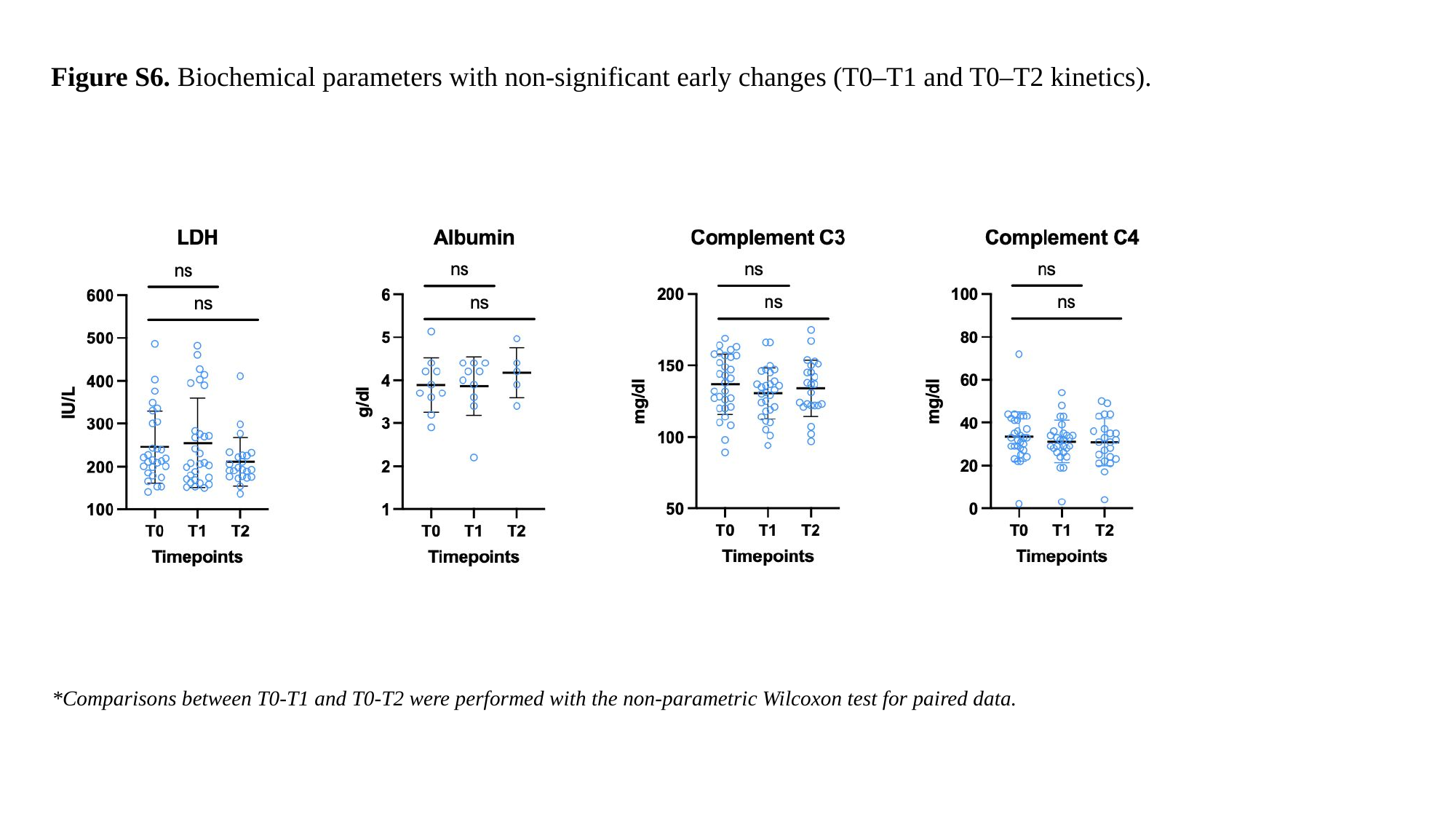

Figure S6. Biochemical parameters with non-significant early changes (T0–T1 and T0–T2 kinetics).
*Comparisons between T0-T1 and T0-T2 were performed with the non-parametric Wilcoxon test for paired data.

## Slide 7
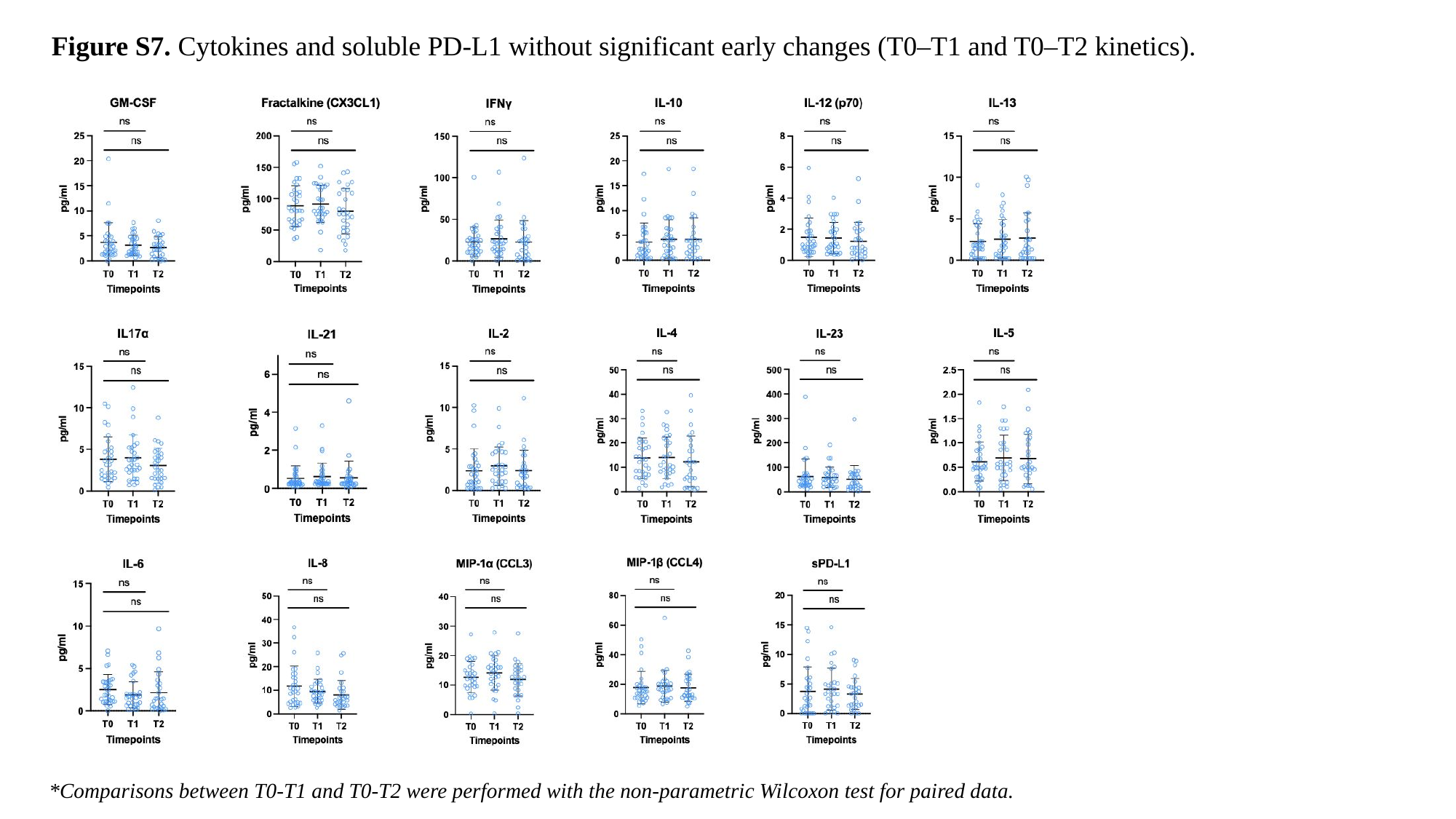

Figure S7. Cytokines and soluble PD-L1 without significant early changes (T0–T1 and T0–T2 kinetics).
*Comparisons between T0-T1 and T0-T2 were performed with the non-parametric Wilcoxon test for paired data.

## Slide 8
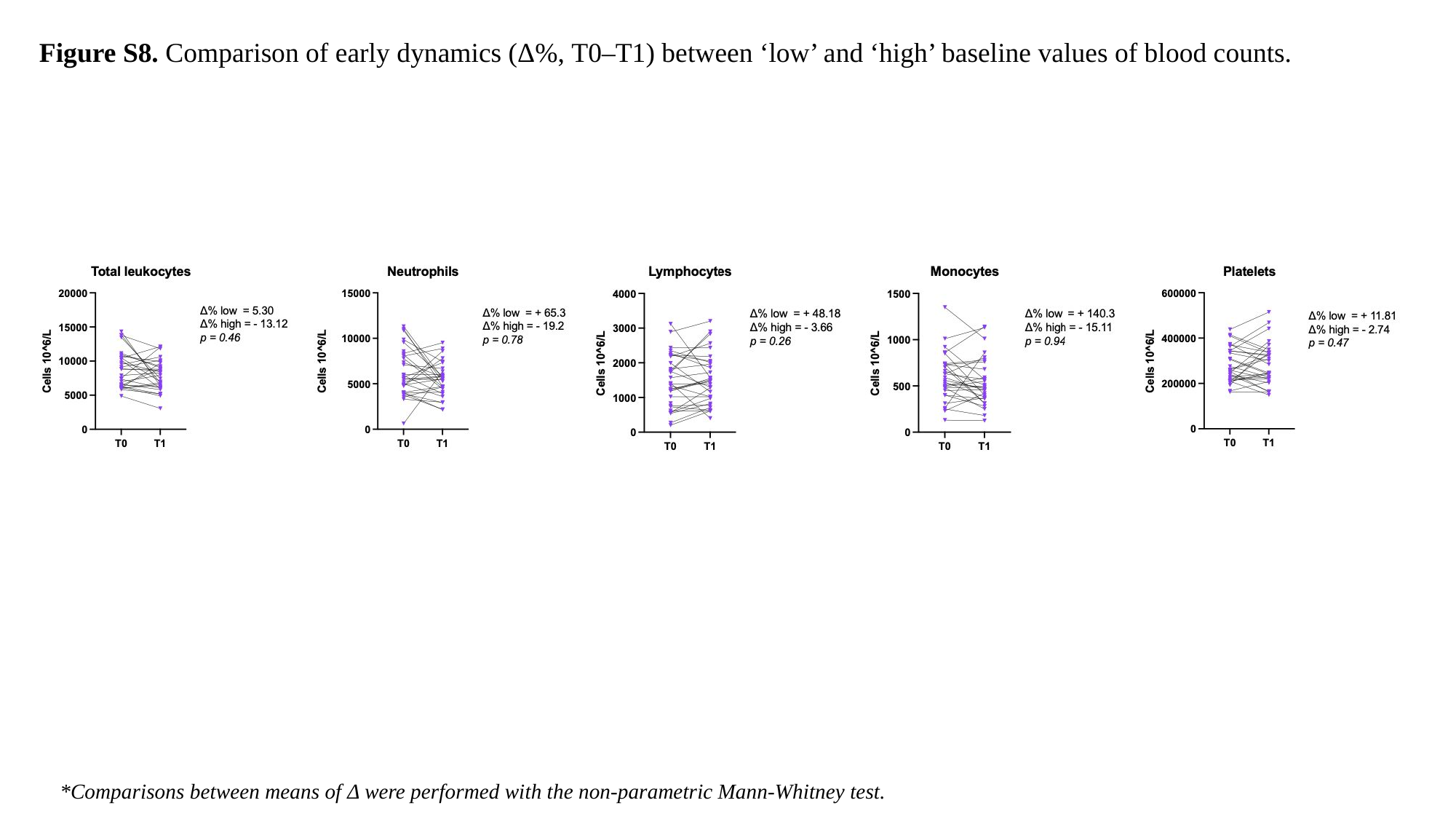

Figure S8. Comparison of early dynamics (Δ%, T0–T1) between ‘low’ and ‘high’ baseline values of blood counts.
*Comparisons between means of Δ were performed with the non-parametric Mann-Whitney test.

## Slide 9
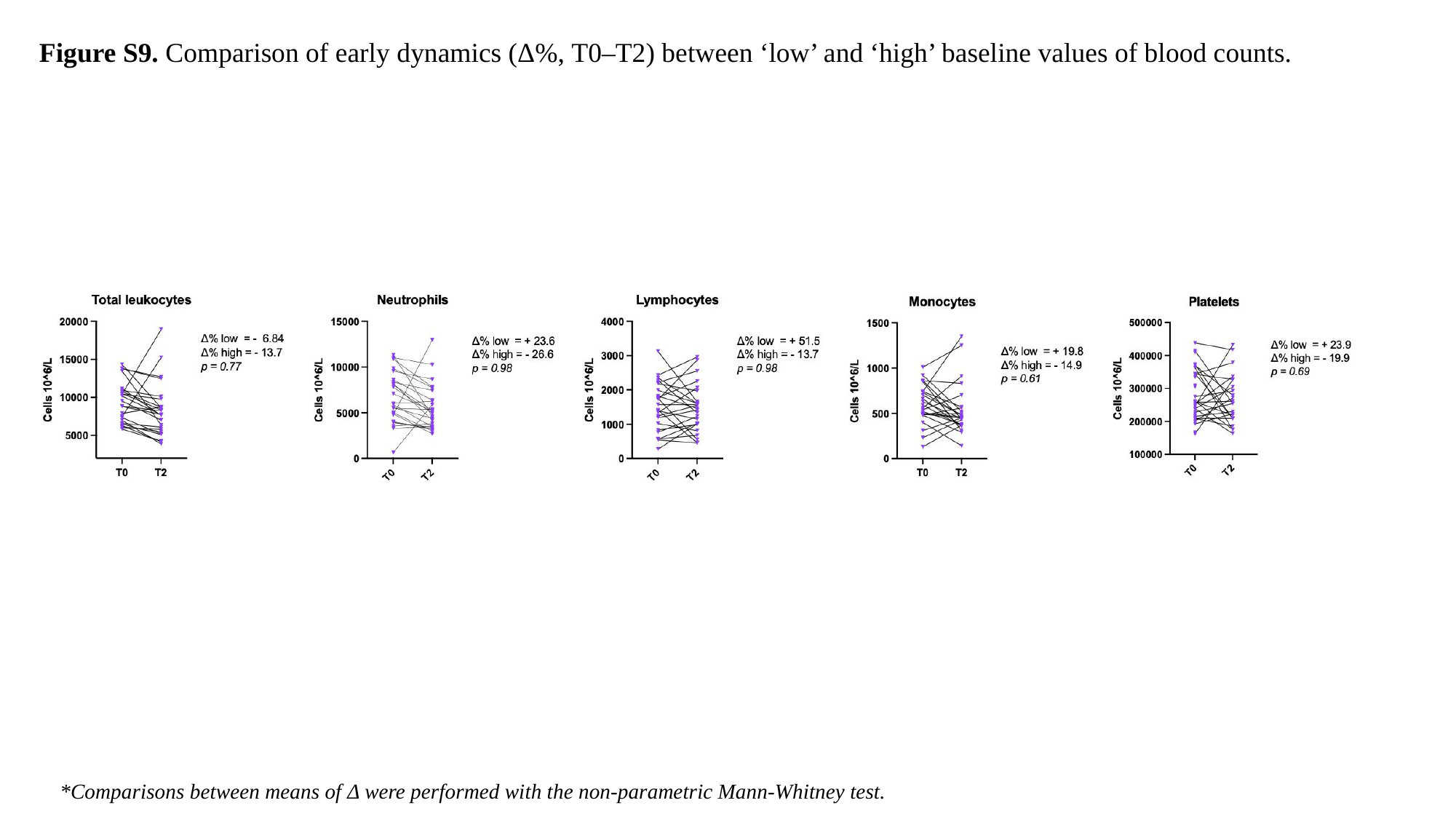

Figure S9. Comparison of early dynamics (Δ%, T0–T2) between ‘low’ and ‘high’ baseline values of blood counts.
*Comparisons between means of Δ were performed with the non-parametric Mann-Whitney test.

## Slide 10
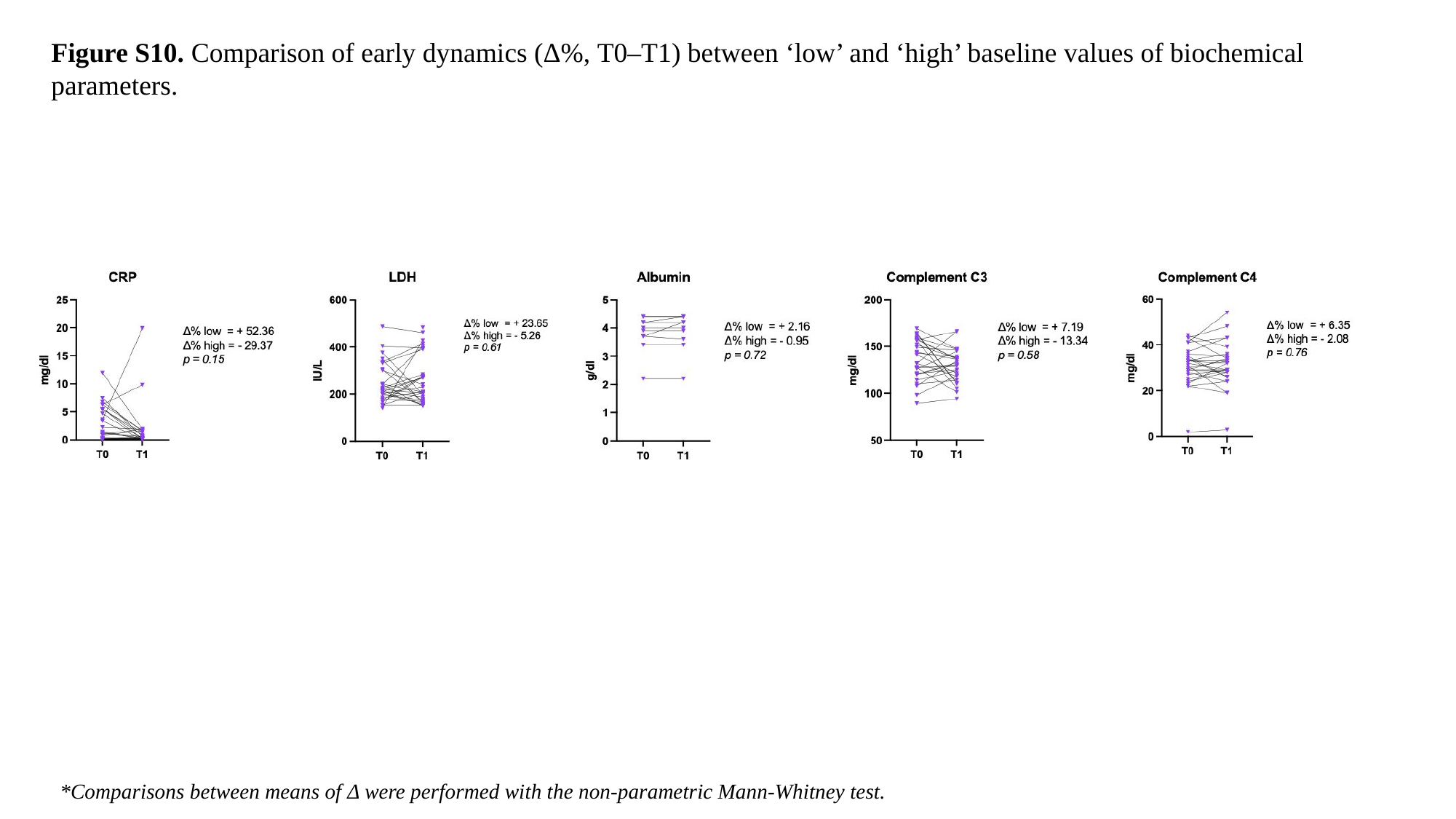

Figure S10. Comparison of early dynamics (Δ%, T0–T1) between ‘low’ and ‘high’ baseline values of biochemical
parameters.
*Comparisons between means of Δ were performed with the non-parametric Mann-Whitney test.

## Slide 11
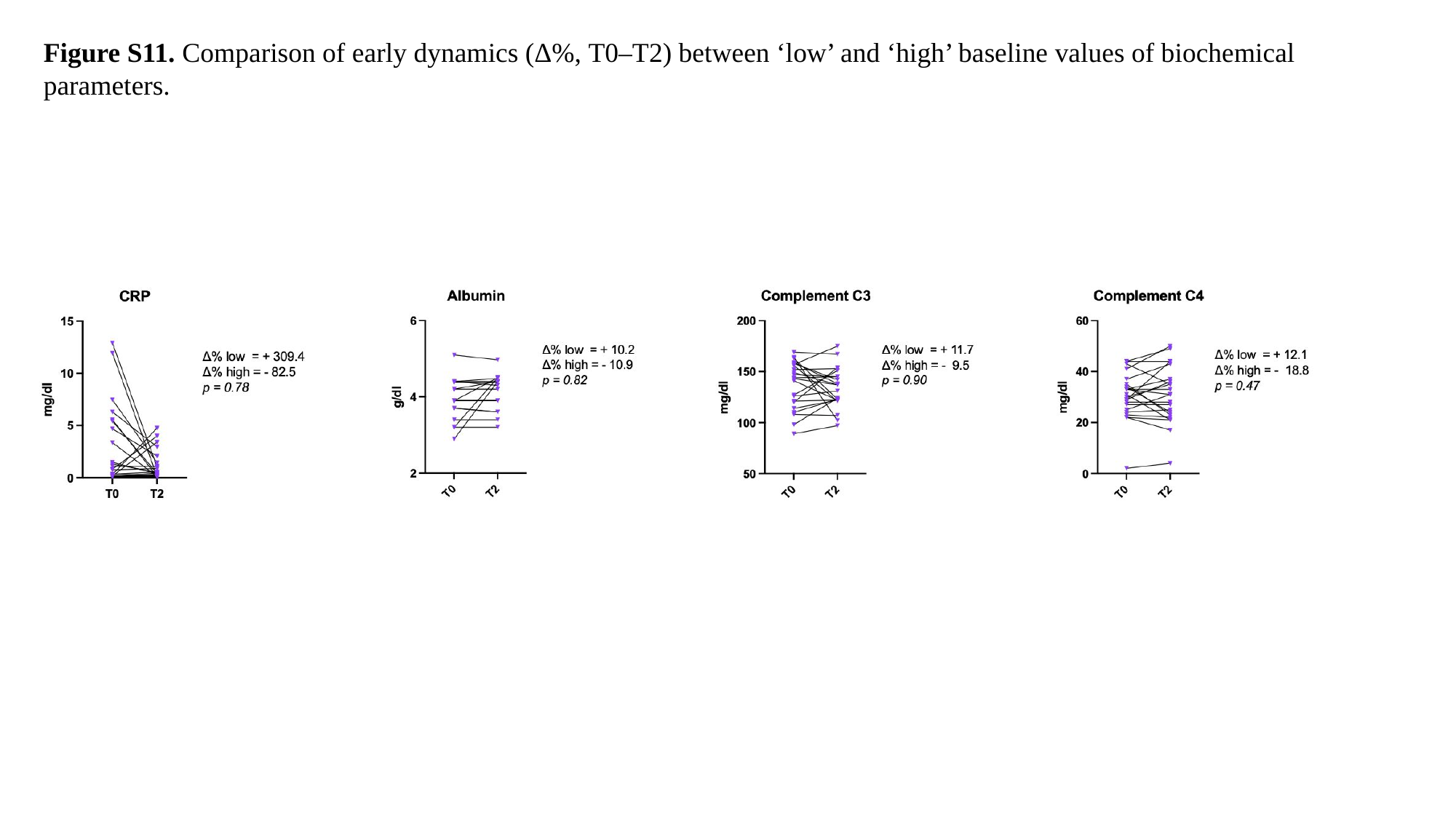

Figure S11. Comparison of early dynamics (Δ%, T0–T2) between ‘low’ and ‘high’ baseline values of biochemical
parameters.

## Slide 12
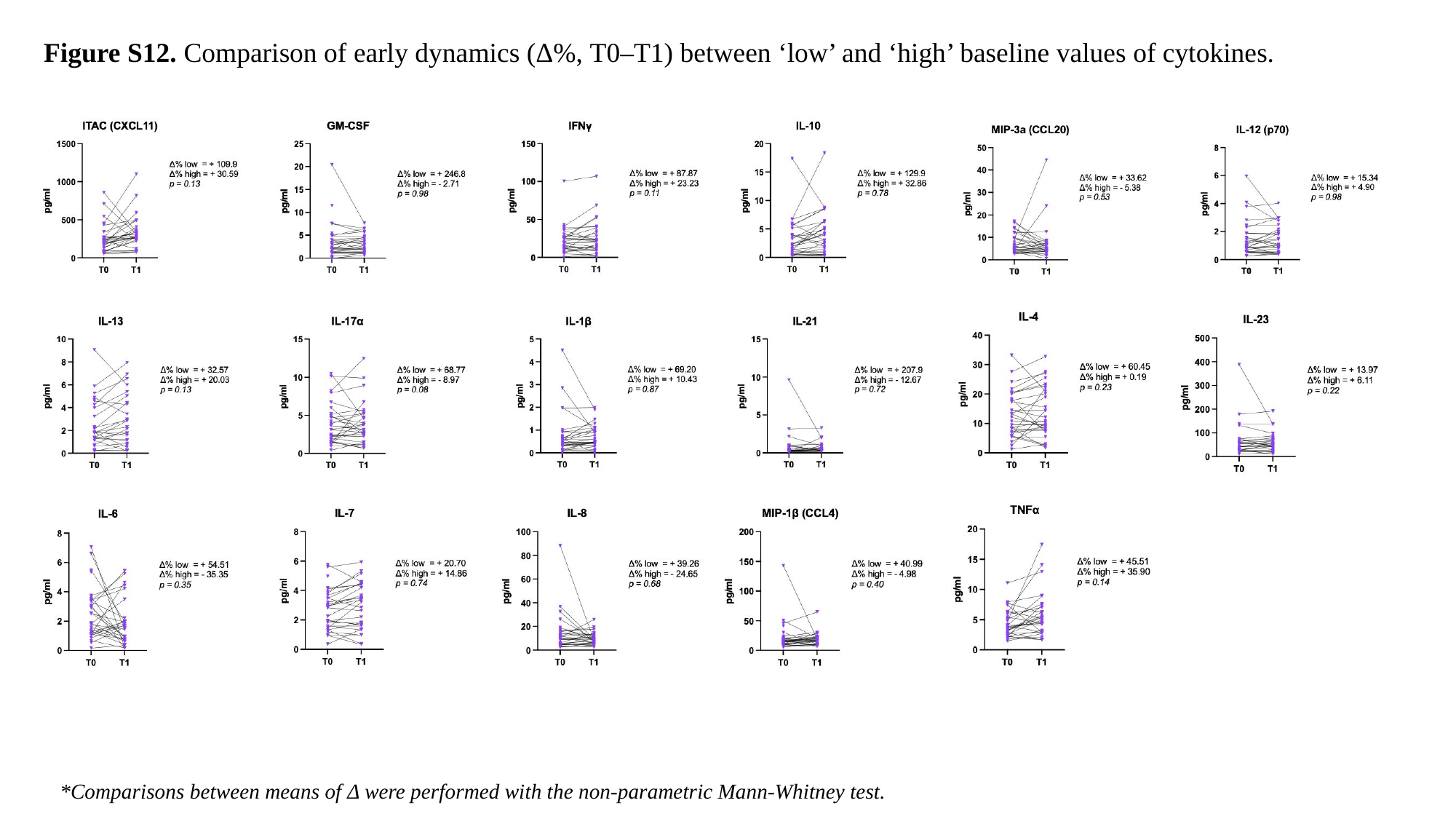

Figure S12. Comparison of early dynamics (Δ%, T0–T1) between ‘low’ and ‘high’ baseline values of cytokines.
*Comparisons between means of Δ were performed with the non-parametric Mann-Whitney test.

## Slide 13
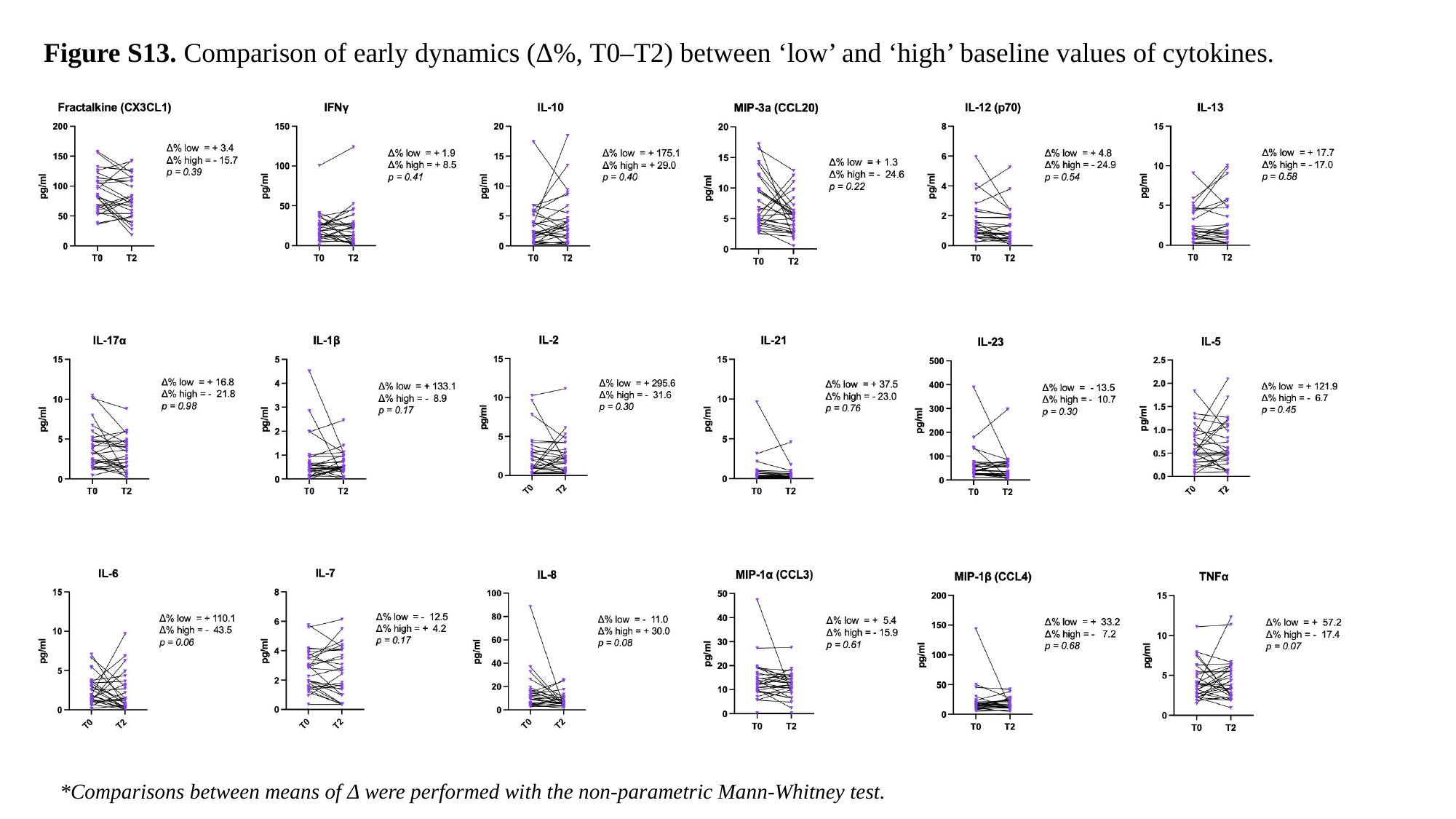

Figure S13. Comparison of early dynamics (Δ%, T0–T2) between ‘low’ and ‘high’ baseline values of cytokines.
*Comparisons between means of Δ were performed with the non-parametric Mann-Whitney test.

## Slide 14
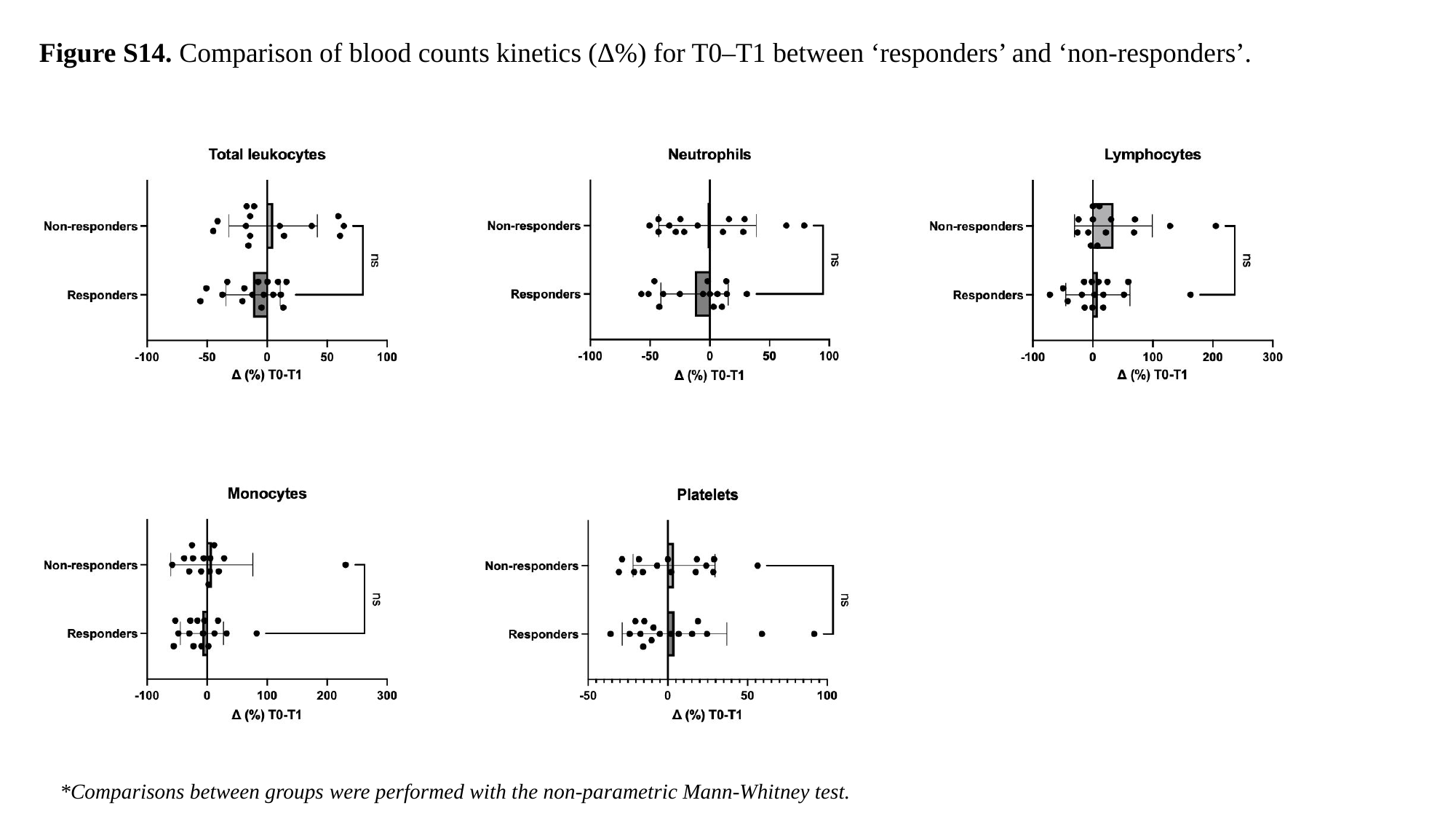

Figure S14. Comparison of blood counts kinetics (Δ%) for T0–T1 between ‘responders’ and ‘non-responders’.
*Comparisons between groups were performed with the non-parametric Mann-Whitney test.

## Slide 15
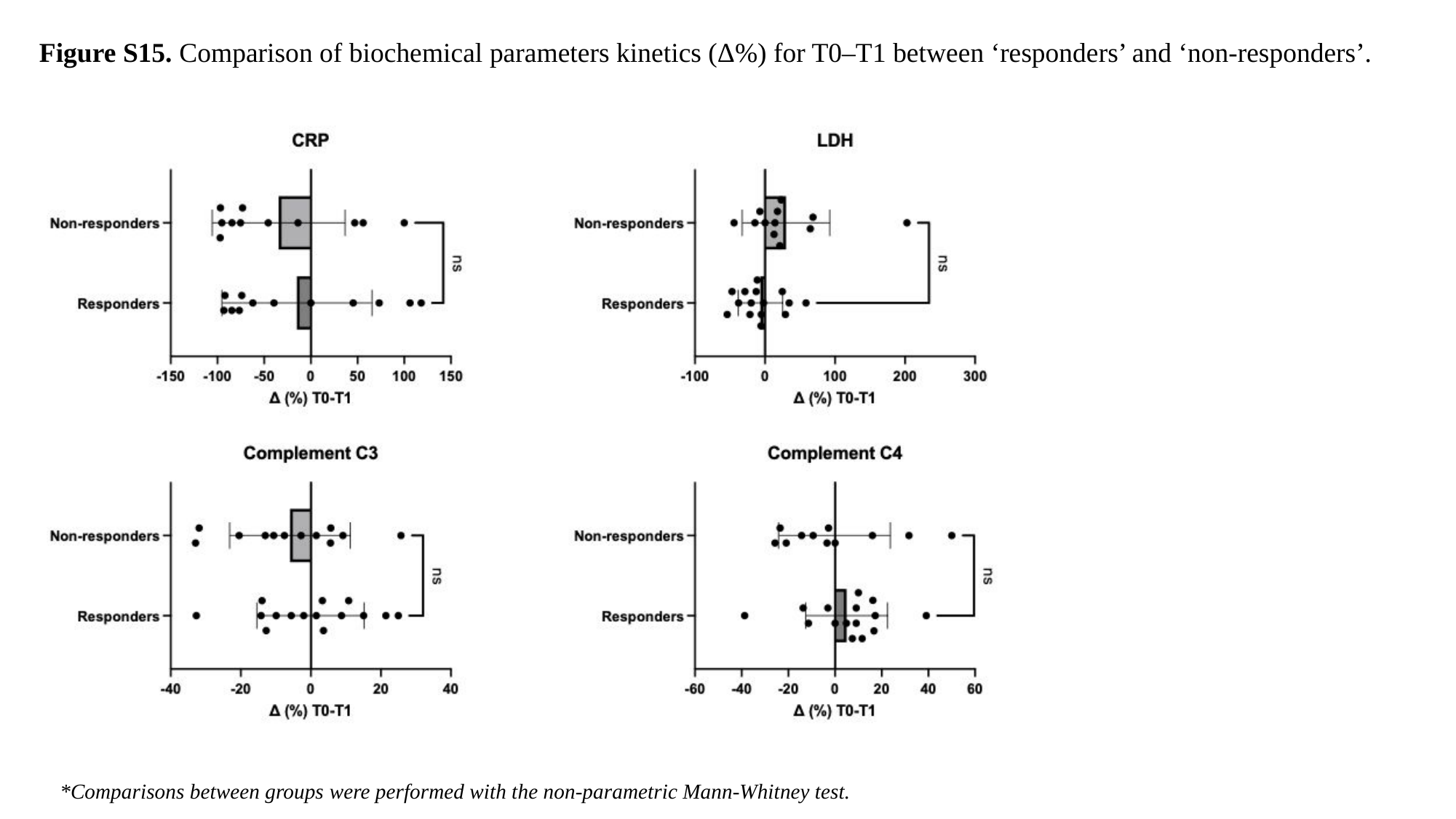

Figure S15. Comparison of biochemical parameters kinetics (Δ%) for T0–T1 between ‘responders’ and ‘non-responders’.
*Comparisons between groups were performed with the non-parametric Mann-Whitney test.

## Slide 16
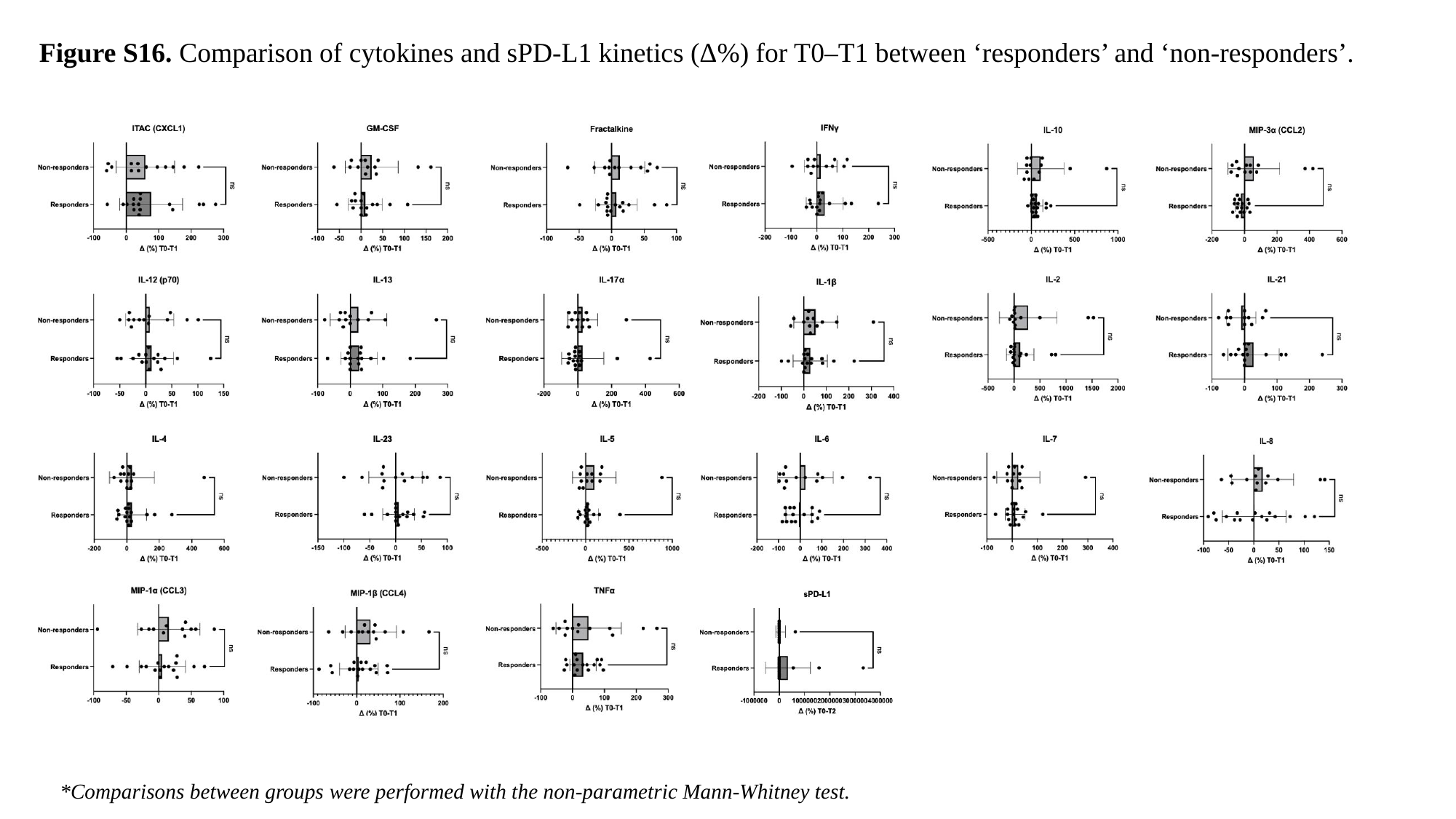

Figure S16. Comparison of cytokines and sPD-L1 kinetics (Δ%) for T0–T1 between ‘responders’ and ‘non-responders’.
*Comparisons between groups were performed with the non-parametric Mann-Whitney test.

## Slide 17
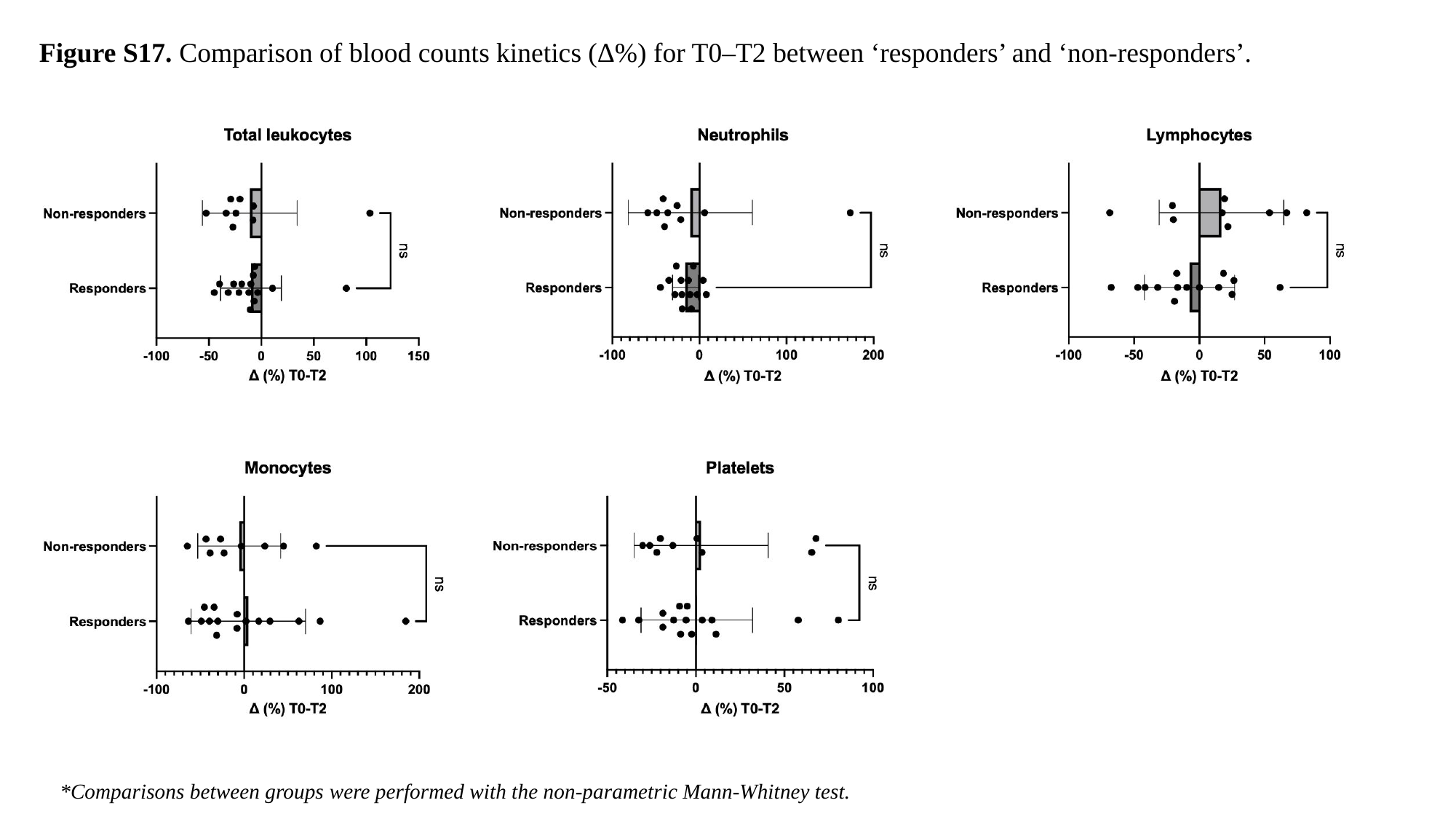

Figure S17. Comparison of blood counts kinetics (Δ%) for T0–T2 between ‘responders’ and ‘non-responders’.
*Comparisons between groups were performed with the non-parametric Mann-Whitney test.

## Slide 18
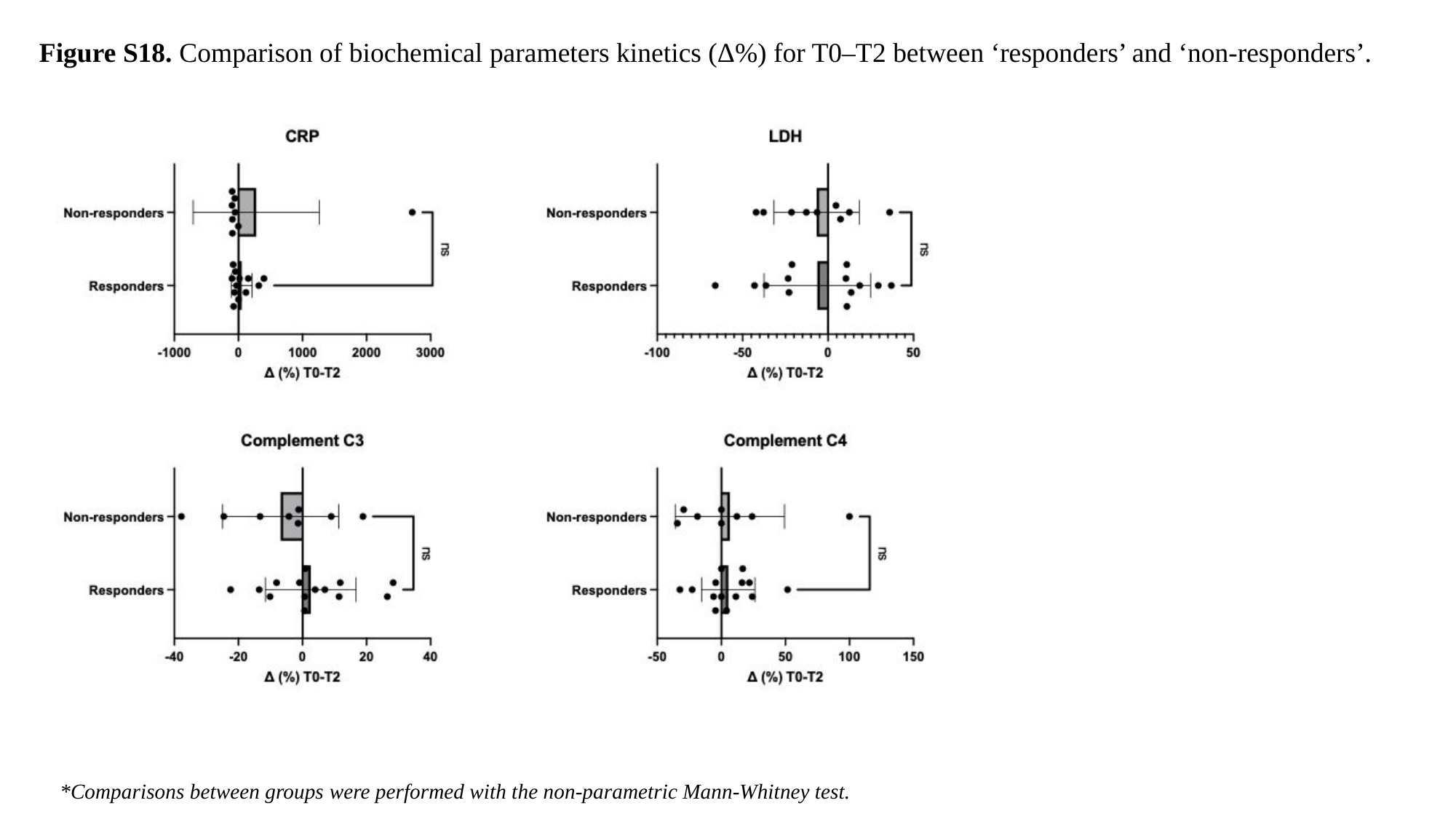

Figure S18. Comparison of biochemical parameters kinetics (Δ%) for T0–T2 between ‘responders’ and ‘non-responders’.
*Comparisons between groups were performed with the non-parametric Mann-Whitney test.

## Slide 19
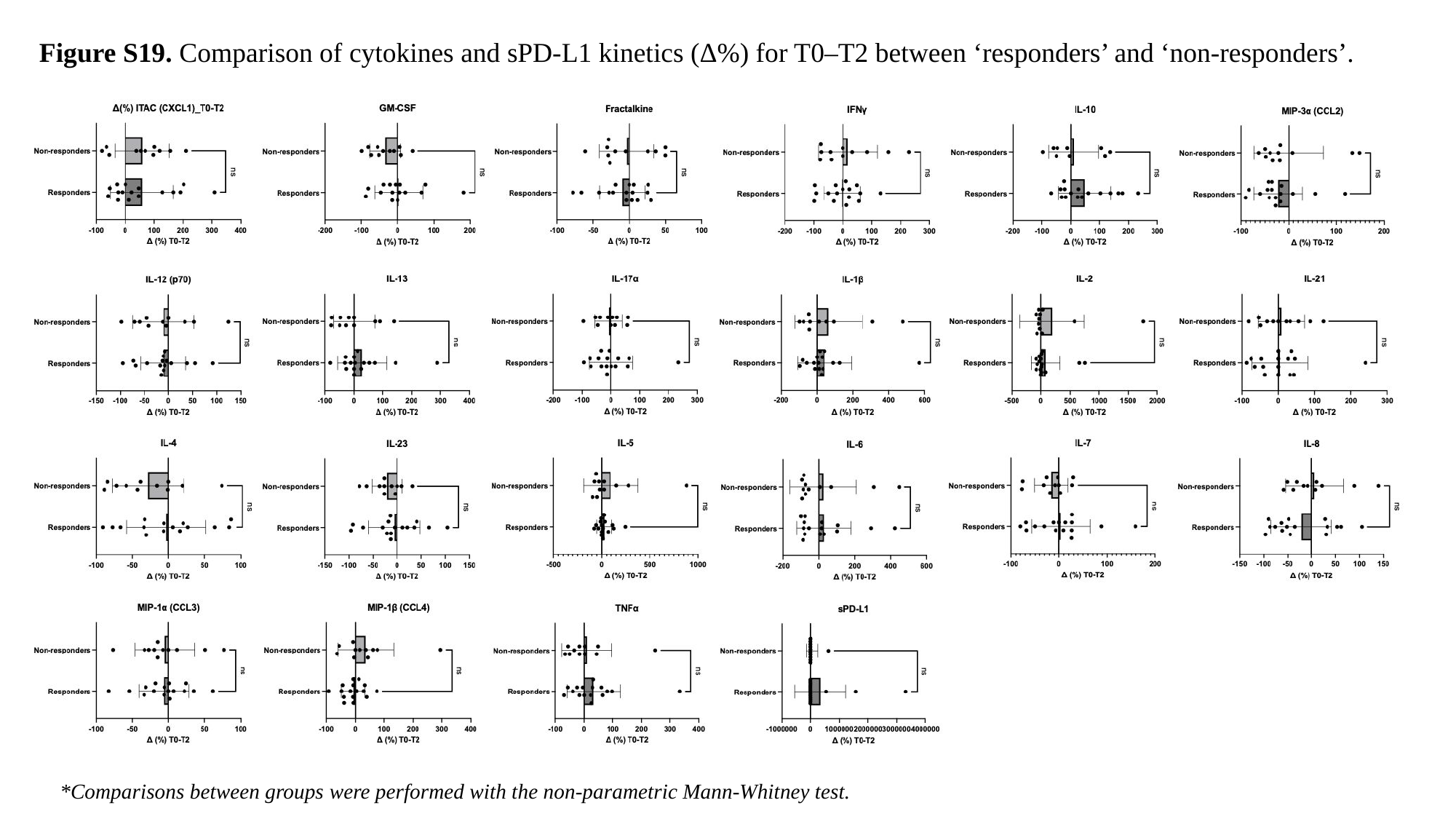

Figure S19. Comparison of cytokines and sPD-L1 kinetics (Δ%) for T0–T2 between ‘responders’ and ‘non-responders’.
*Comparisons between groups were performed with the non-parametric Mann-Whitney test.

## Slide 20
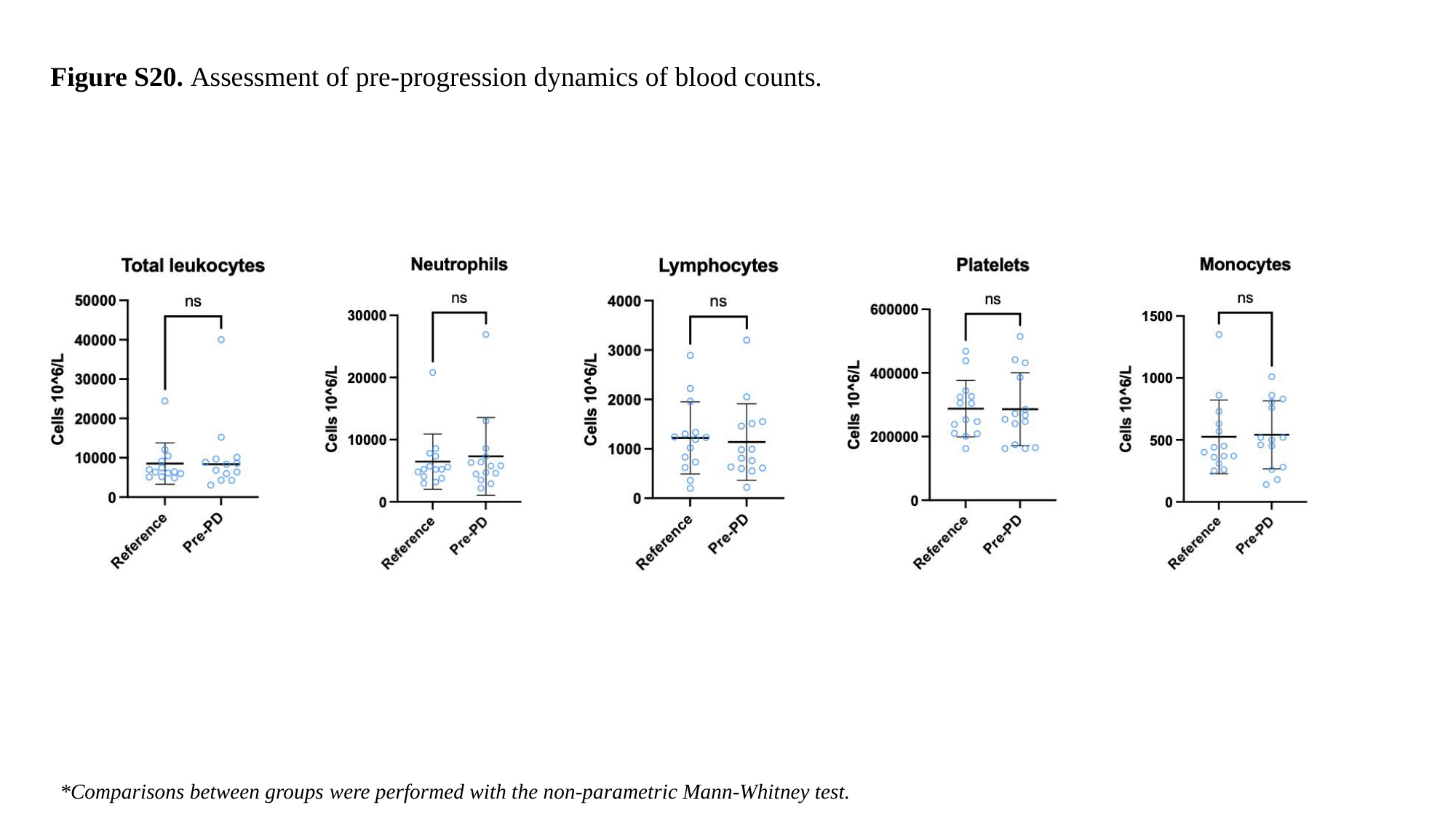

Figure S20. Assessment of pre-progression dynamics of blood counts.
*Comparisons between groups were performed with the non-parametric Mann-Whitney test.

## Slide 21
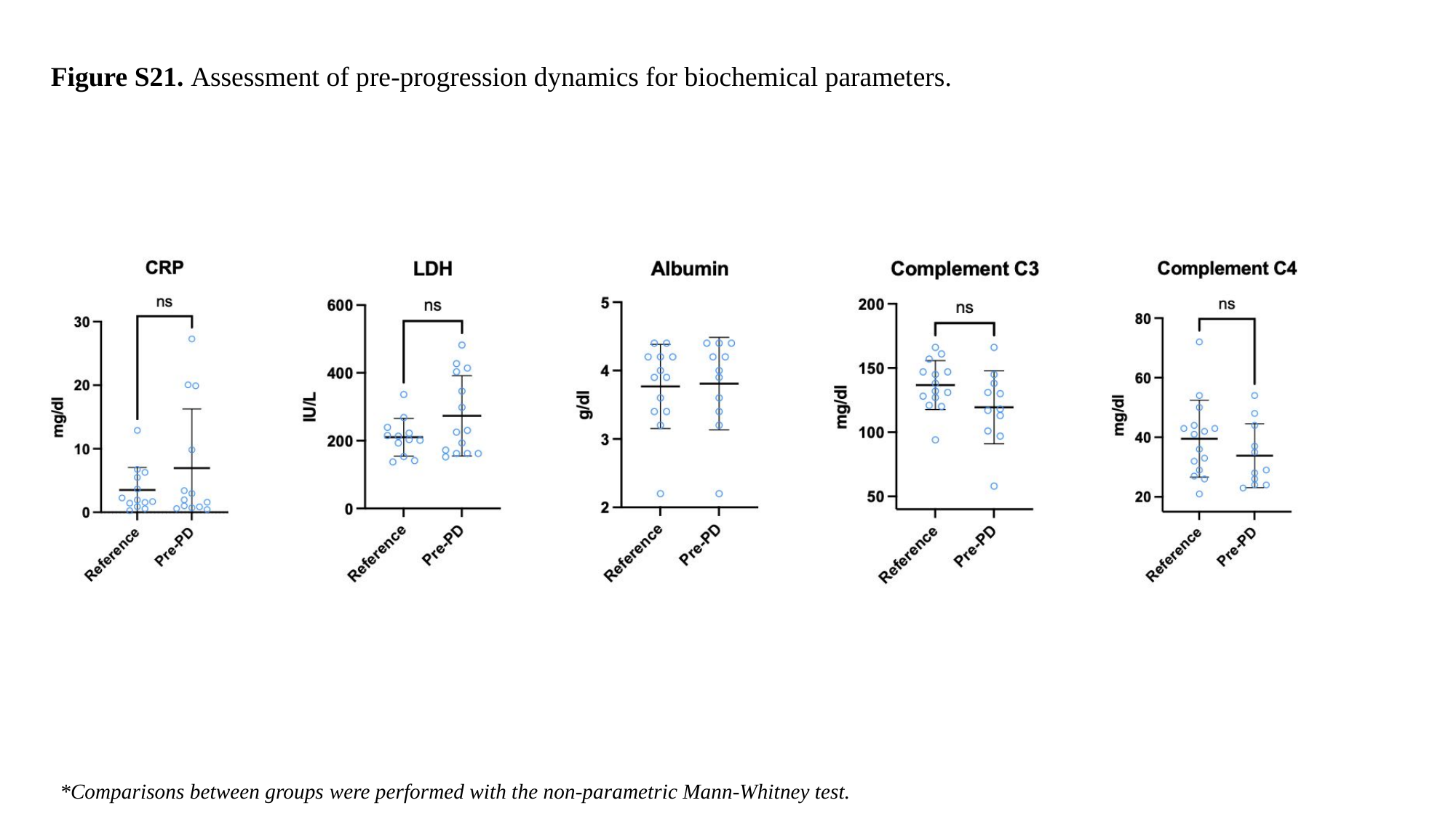

Figure S21. Assessment of pre-progression dynamics for biochemical parameters.
*Comparisons between groups were performed with the non-parametric Mann-Whitney test.

## Slide 22
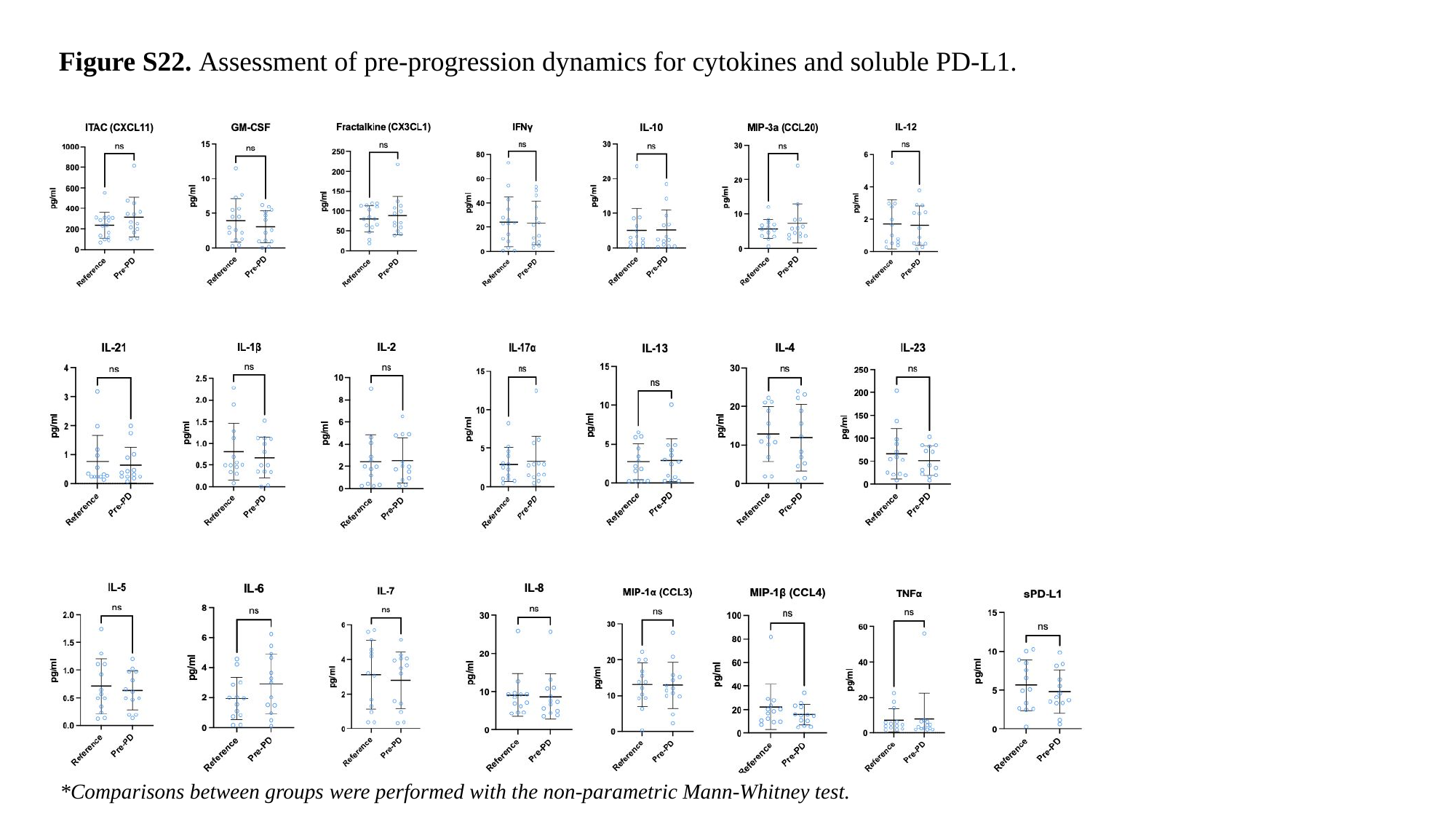

Figure S22. Assessment of pre-progression dynamics for cytokines and soluble PD-L1.
*Comparisons between groups were performed with the non-parametric Mann-Whitney test.
