# Supplementary material for: In-Treatment Kinetics of Peripheral Blood Immune Markers in PD-L1 High Non-Small Cell Lung Cancer and Prognostic Relevance for Immunotherapy Outcomes
Source: Cancers (Basel). 2026 May 17;18(10):1623. doi: 10.3390/cancers18101623 (PMC13204403; doi:10.3390/cancers18101623)
Supplement: Supplementary file 1 [file cancers-18-01623-s001.zip › Table S1-7.pdf]

Table S1. Summary of the cytokines/chemokines analyzed with the MILLIPLEX® MAP Human High Sensitivity T Cell Panel and their lower limits of detection (LOD).

Cytokine/Chemokine	LOD (pg/mL)
**I-TAC (CXCL11)**	1.45
**GM-CSF**	1.21
**Fractalkine (CX3CL1)**	17.93
**IFN-γ**	0.47
**IL-10**	1.46
**MIP-3α (CCL20)**	0.61
**IL-12 (p70)**	0.49
**IL-13**	0.20
**IL-17α (CTLA-8)**	0.71
**IL-1β**	0.49
**IL-2**	0.42
**IL-21**	0.24
**IL-4**	1.60
**IL-5**	0.49
**IL-23**	7.80
**IL-6**	0.16
**IL-7**	0.35
**IL-8 (CXCL8)**	0.30
**MIP-1α (CCL3)**	6.76
**MIP-1β (CCL4)**	3.84
**TNF-α**	0.43

CCL: C-C motif chemokine ligand; CTLA: cytotoxic T-lymphocyte-associated protein; CXCL: C-X-C motif chemokine ligand; IFN-γ: interferon-γ; IL: interleukin; I-TAC: interferon-inducible T-cell alpha chemoattractant; LOD: limit of detection; MIP: macrophage inflammatory protein; TNF-α: tumor necrosis factor-α.

Table S2. Baseline clinical and pathological characteristics.

Baseline characteristics	Number, n (%)
**Patients enrolled**	31 (100.0)
**Age***	72 (53, 83)
**Sex (male)**	23 (74.2)
**NSCLC histology**	31 (100.0)
**Histological type**	
Adenocarcinoma	22 (71.0)
Squamous carcinoma	9 (29.0)
**Stage at treatment initiation**	
III	4 (12.9)
IV	27 (87.1)
**ECOG PS**	
0	11 (35.5)
1	11 (35.5)
≥2	9 (29.0)
**Smoking status**	
Former	22 (71.0)
Current	9 (29.0)
**Pack–years***	70 (10, 150)
**PD-L1 (TPS, %)***	80 (50, 100)
**Actionable mutations (KRAS G12C)**	2 (6.5)
**Radiotherapy (at baseline or within 2 months)**	18 (58.1)
Brain	7 (22.6)
Thorax	7 (22.6)
Other (palliative)	4 (12.9)
**Immunomodulatory agents at baseline**	14 (45.2)

BSA: body surface area; ECOG PS: Eastern Cooperative Oncology Group performance status; PD-L1: programmed-death ligand-1; TPS: tumor proportion score; *: median (IQR); ¶: mean (standard deviation); NSCLC: non-small cell lung cancer.

Table S3. Response and survival outcomes at the 2-year follow-up.

Clinical outcomes	Number, n (%)
**Pembrolizumab monotherapy**	31 (100.0)
**BOR (RECIST)**	
CR	1 (3.2)
PR	15 (48.4)
SD	7 (22.6)
PD	8 (25.8)
**ORR**	51.6 (n=16)
**Time of response**	
3 months	14 (45.2)
6 months	2 (6.5)
**Sustained response at 2 years**	12 (38.7)
**Clinical benefit**	
6 months	19 (61.3)
1 year	17 (54.8)
2 years	14 (45.2)
**Alive at 2 years**	21 (67.7)
**Median PFS at 2 years (months)**	17.9
**Median survival at 2 years (months)**	NR

BOR: best objective response; CR: complete response; PFS: progression-free survival; NR: not reached; ORR: objective response rate; PD: progressive disease; PR: partial response; RECIST: Response Evaluation Criteria in Solid Tumors; SD: stable disease.

Table S4. Comparison of baseline clinicopathological characteristics between patients who completed (completers) the 1-year follow-up and those who did not (non-completers).

Baseline characteristics	Completers (n, %)	Non-completers (n, %)	p-Value
**Age >65**	16 (80)	9 (82)	1.0
**Gender male**	16 (80)	7 (64)	0.4
**Poor PS (≥2)**	5 (25)	4 (36)	0.7
**Stage III**	2 (10)	2 (18)	0.6
**Adenocarcinoma histology**	16 (80)	6 (55)	0.2
**Active smoker**	5 (25)	4 (36)	0.4
**Radiotherapy**	7 (35)	6 (55)	0.5
**Corticosteroids**	8 (40)	6 (55)	0.5

*Comparisons were performed with Fisher's exact test.

Table S5. Association between the pretreatment/baseline (T0) values and the significant early changes (Δ%, T0– T1 and T0–T2) of the peripheral blood immune markers with 6-month clinical benefit (CB6), assessed with univariate logistic regression.

Parameter	Odds ratio (OR)	95%CI	p-Value	AUC (95%CI)
**WBCs_T0**	1.00	0.99 – 1.00	0.95	0.62 (0.40 – 0.85)
**Neu_T0**	0.99	0.99 – 1.00	0.58	0.54 (0.31 – 0.76)
**Ly_T0**	0.99	0.99 – 1.00	0.42	0.59 (0.38 – 0.81)
**Mono_T0**	1.00	0.99 – 1.00	0.99	0.57 (0.32 – 0.81)
**Plts_T0**	1.00	1.00 – 1.00	0.85	0.52 (0.30 – 0.74)
**NLR_T0**	0.97	0.87 – 1.08	0.59	0.59 (0.38 – 0.81)
**dNLR_T0**	0.87	0.59 – 1.25	0.45	0.59 (0.36 – 0.81)
**LMR_T0**	1.12	0.87 – 1.72	0.42	0.55 (0.33 – 0.76)
**PLR_T0**	0.99	0.99 – 1.00	0.36	0.56 (0.34 – 0.79)
**SII**	1.00	0.99 – 1.00	0.68	0.58 (0.36 0 0.80)
**CRP_T0**	0.81	0.63 – 0.98	0.02	0.77 (0.60 – 0.94)
**LDH_T0**	1.00	0.99 – 1.00	0.53	0.57 (0.35 – 0.79)
**Alb_T0**	2.21	0.57 – 10.52	0.25	0.60 (0.39 – 0.82)

**CAR**	0.55	0.24 – 1.02	0.06	0.77 (0.59 – 0.95)
**LIPI**	0.97	0.35 – 2.70	0.95	0.51 (0.30 – 0.72)
**C3_T0**	1.00	0.97 – 1.00	0.69	0.53 (0.31 – 1.05)
**C4_T0**	0.85	0.72 – 0.95	< 0.01	0.80 (0.65 – 0.96)
**C3/C4 ratio**	4.43	1.59 – 18.74	< 0.01	0.82 (0.67 – 0.97)
**ITAC (CXCL11)_T0**	1.00	0.99 – 1.00	0.56	0.58 (0.37 – 0.79)
**GM-CSF_T0**	0.84	0.62 – 1.03	0.11	0.55 (0.31 – 0.79)
**Fractalkine (CX3CL1)_T0**	1.00	0.98 – 1.03	0.78	0.53 (0.31 – 0.75)
**IFNγ_T0**	1.00	0.97 – 1.06	0.70	0.51 (0.28 – 0.74)
**IL-10_T0**	0.85	0.66 – 1.04	0.13	0.55 (0.32 – 0.79
**MIP-3α (CCL20)_T0**	1.04	0.87 – 1.28	0.65	0.54 (0.33 – 0.75)
**IL-12 (p70)_T0**	0.76	0.39 – 1.40	0.35	0.52 (0.28 – 0.76)
**IL-13_T0**	0.78	0.52 – 1.12	0.18	0.61(0.39 – 0.84)
**IL-17α_T0**	0.95	0.77 – 1.20	0.60	0.60 (0.35 – 0.84)
**IL-1β_T0**	0.73	0.29 – 1.66	0.44	0.52 (0.28 – 0.75)
**IL-2_T0**	0.99	0.75 – 1.36	0.96	0.61 (0.38 – 0.83)
**IL-21_T0**	0.70	0.25 – 1.14	0.17	0.54 (0.32 – 0.77)
**IL-4_T0**	0.99	0.91 – 1.09	0.89	0.51 (0.29 – 0.72)
**IL-23_T0**	0.99	0.98 – 1.00	0.34	0.52 (0.30 – 0.75)
**IL-5_T0**	0.63	0.09 – 4.27	0.63	0.51 (0.28 – 0.75)
**IL-6_T0**	0.58	0.31 – 0.92	0.02	0.68 (0.45 – 0.90)
**IL-7_T0**	0.75	0.41 – 1.29	0.30	0.61 (0.30 – 0.82)
**IL-8_T0**	1.02	0.97 – 1.10	0.57	0.57 (0.37 – 0.80)
**MIP-1α (CCL3)_T0**	1.05	0.95 – 1.20	0.39	0.56 (0.34 – 0.78)
**MIP-1β (CCL4)_T0**	1.02	0.98 – 1.09	0.45	0.52 (0.31 – 0.73)
**TNFα_T0**	0.82	0.56 – 1.17	0.27	0.69 (0.49 – 0.88)
**sPD-L1_T0**	1.04	0.87 – 1.30	0.67	0.53 (0.31 – 0.75)
**Δ% (T0–T2) WBCs**	0.99	0.96 – 1.02	0.51	0.59 (0.21 – 0.96)
**Δ% (T0–T2) Neu**	0.99	0.99 – 1.00	0.98	0.56 (0.17 – 0.96)
**Δ% (T0–T1) CRP**	0.99	0.99 – 1.00	0.41	0.64 (0.42 – 0.86)
**Δ% (T0–T2) CRP**	1.01	0.99 – 1.00	0.30	0.59 (0.32 – 0.96)
**Δ% (T0–T2) LDH**	1.03	0.99 – 1.10	0.21	0.69 (0.45 – 0.93)
**Δ% (T0–T1) ITAC (CXCL11)**	1.00	0.99 – 1.02	0.40	0.57 (0.35 – 0.79)
**Δ% (T0–T2) ITAC (CXCL11)**	0.99	0.98 – 1.00	0.65	0.53 (0.29 – 0.78)
**Δ% (T0–T2) GM-CSF**	0.99	0.98 – 1.00	0.35	0.59 (0.33 – 0.86)
**Δ% (T0–T1) Fractalkine (CX3CL1)**	1.01	0.98 – 1.04	0.44	0.55 (0.30 – 0.80)
**Δ% (T0–T2) MIP-3α (CCL20)**	0.99	0.98 – 1.01	0.52	0.64 (0.42 – 0.87)
**Δ% (T0–T1) IL-17α**	1.00	0.99 – 1.00	0.99	0.57 (0.31 – 0.83)
**Δ% (T0–T1) IL-1β**	0.99	0.98 – 1.00	0.05	0.62 (0.38 – 0.87)
**Δ% (T0–T1) IL-2**	0.99	0.99 – 1.00	0.64	0.54 (0.29 – 0.78)
**Δ% (T0–T2) IL-4**	0.99	0.98 – 1.00	0.66	0.56 (0.30 – 0.83)
**Δ% (T0–T1) IL-5**	0.99	0.99 – 1.00	0.19	0.56 (0.29 – 0.83)
**Δ% (T0–T1) IL-7**	1.02	0.99 – 1.05	0.13	0.67 (0.45 – 0.89)
**Δ% (T0–T1) MIP-1α (CCL3)**	1.00	0.99 – 1.02	0.47	0.53 (0.26 – 0.80)
**Δ% (T0–T1) TNFα**	1.00	0.99 – 1.02	0.71	0.62 (0.36 – 0.87)
**Δ% (T0–T1) sPD-L1**	1.00	1.00 – 1.00	0.40	0.59 (0.38 – 0.81)
**Δ% (T0–T2) sPD-L1**	1.00	0.99 – 1.00	0.25	0.67 (0.43 – 0.91)

Alb: albumin; AUC: area under the curve; CAR: CRP-to-albumin ratio; CCL3, 4, 20: C-C motif chemokine ligand; CI: confidence interval; CRP: C-reactive protein; CXCL11: C-X-C motif chemokine ligand 11; CX3CL1: C-X3-C motif chemokine ligand 1; C3/4: complement proteins; C3/C4: C3-to-C4 ratio; dNLR: derived NLR; IFN-γ: interferon-γ; IL: interleukin; ITAC: interferon-inducible T-cell alpha chemoattractant; LDH: lactate dehydrogenase; LIPI: Lung Immune Prognostic Index; LMR: lymphocyte-to-monocyte ratio; Ly: lymphocytes; MIP-3α: macrophage inflammatory protein α; Mono: monocytes; Neu: neutrophils; NLR: neutrophil-to-lymphocyte ratio; PLR: platelet-to-lymphocyte ratio; Plts: platelets; SII: Systemic Inflammation Index; sPD-L1: soluble programmed-death ligand-1; T0: baseline/pretreatment sampling; T1: 21 days (before cycle 2); T2: 3 months; WBCs: white blood cells; Δ%: percentage change.

Table S6. Association between the pretreatment/baseline (T0) values and the significant early changes (Δ%, T0–T1 and T0–T2) of the peripheral blood immune markers with progression-free survival at 2 years (2y PFS), assessed with univariate Cox regression.

Parameter	Hazard ratio (HR)	95%CI	p-Value	Harrell's C-index (95%CI)
**WBCs_T0**	1.00	0.99 – 1.00	0.69	0.45 (0.31 – 0.60)
**Neu_T0**	1.12	0.99 – 1.00	0.26	0.52 (0.38 – 0.67)
**Ly_T0**	0.99	0.99 – 1.00	0.45	0.55 (0.37 – 0.73)
**Mono_T0**	1.00	0.99 – 1.00	0.83	0.50 (0.33 – 0.66)
**Plts_T0**	1.00	1.00 – 1.00	0.39	0.55 (0.40 – 0.69)
**NLR_T0**	1.01	0.94 – 1.06	0.63	0.59 (0.44 – 0.75)
**dNLR_T0**	1.09	0.87 – 1.28	0.39	0.58 (0.42 – 0.75)
**LMR_T0**	0.99	0.82 – 1.11	0.91	0.56 (0.40 – 0.72)
**PLR_T0**	1.00	0.99 – 1.00	0.46	0.54 (0.37 – 0.72)
**SII**	1.00	0.99 – 1.00	0.91	0.57 (0.41 – 0.73)
**CRP_T0**	1.13	1.01 – 1.24	0.01	0.70 (0.58 – 0.79)
**LDH_T0**	0.99	0.99 – 1.00	0.93	0.52 (0.37 – 0.68)
**Alb_T0**	0.56	-1.16 – 0.68	0.56	0.54 (0.38 – 0.70)
**CAR**	1.45	0.99 – 20.2	0.03	0.71 (0.56 – 0.79)
**LIPI**	1.41	0.73 – 2.70	0.30	0.56 (0.43 – 0.68)
**C3_T0**	0.99	0.98 – 1.02	0.89	0.51 (0.35 – 0.67)
**C4_T0**	1.09	1.03 – 1.50	< 0.01	0.71 (0.59 – 0.83)
**C3/C4 ratio**	0.52	0.29 – 0.86	0.02	0.72 (0.60 – 0.83)
**ITAC (CXCL11)_T0**	1.00	0.99 – 1.00	0.38	0.50 (0.39 – 0.62)
**GM-CSF_T0**	1.07	0.95 – 1.17	0.18	0.50 (0.35 – 0.66)
**Fractalkine (CX3CL1)_T0**	0.99	0.98 – 1.01	0.72	0.51 (0.36 – 0.66)
**IFNγ_T0**	1.00	0.97 – 1.02	0.87	0.50 (0.33 – 0.67)
**IL-10_T0**	1.21	1.03 – 1.40	0.01	0.59 (0.52 – 0.76)
**MIP-3α (CCL20)_T0**	0.99	0.87 – 1.11	0.91	0.50 (0.33 – 0.66)
**IL-12 (p70)_T0**	1.33	0.92 – 1.80	0.09	0.55 (0.40 – 0.69)
**IL-13_T0**	1.14	0.91 – 1.39	0.21	0.57 (0.41 – 0.73)
**IL-17α_T0**	1.07	0.89 – 1.24	0.41	0.44 (0.27 – 0.61)
**IL-1β_T0**	1.12	0.61 – 1.71	0.66	0.49 (0.34 – 0.64)
**IL-2_T0**	1.05	0.86 – 1.23	0.57	0.47 (0.33 – 0.62)
**IL-21_T0**	1.15	0.89 – 1.36	0.18	0.55 (0.41 – 0.70)
**IL-4_T0**	1.00	0.94 – 1.06	0.98	0.49 (0.34 – 0.64)
**IL-23_T0**	1.00	0.99 – 1.00	0.41	0.48 (0.33 – 0.62)
**IL-5_T0**	2.65	0.67 – 9.62	0.15	0.56 (0.42 – 0.71)
**IL-6_T0**	1.63	1.20 – 2.24	< 0.01	0.72 (0.54 – 0.81)
**IL-7_T0**	1.15	0.78 – 1.66	0.48	0.55 (0.38 – 0.71)
**IL-8_T0**	0.98	0.92 – 1.01	0.32	0.49 (0.34 – 0.64)
**MIP-1α (CCL3)_T0**	0.96	0.89 – 1.02	0.30	0.55 (0.42 – 0.69)
**MIP-1β (CCL4)_T0**	0.99	0.94 – 1.00	0.39	0.49 (0.33 – 0.65)
**TNFα_T0**	1.10	0.90 – 1.31	0.29	0.62 (0.48 – 0.76)
**sPD-L1_T0**	1.05	0.94 – 1.16	0.34	0.58 (0.45 – 0.70)
**Δ% (T0–T2) WBCs**	0.99	0.95 – 1.01	0.60	0.68 (0.46 – 0.89)
**Δ% (T0–T2) Neu**	0.99	0.99 – 1.00	0.62	0.54 (0.31 – 0.77)
**Δ% (T0–T1) CRP**	1.00	0.99 – 1.00	0.20	0.54 (0.39 – 0.71)
**Δ% (T0–T2) CRP**	0.99	0.98 – 1.00	0.39	0.57 (0.38 – 0.76)
**Δ% (T0–T2) LDH**	1.01	0.98 – 1.02	0.96	0.48 (0.26 – 0.69)
**Δ% (T0–T1) ITAC (CXCL11)**	0.99	0.99 – 1.00	0.42	0.54 (0.40 – 0.68)
**Δ% (T0–T2) ITAC (CXCL11)**	1.03	0.99 – 1.01	0.27	0.56 (0.30 – 0.73)
**Δ% (T0–T2) GM-CSF**	0.99	0.99 – 1.00	0.31	0.49 (0.34 – 0.64)
**Δ% (T0–T1) Fractalkine (CX3CL1)**	0.99	0.98 – 1.00	0.38	0.55 (0.41 – 0.69)
**Δ% (T0–T2) MIP-3α (CCL20)**	1.00	0.99 – 1.00	0.15	0.60 (0.45 – 0.75)
**Δ% (T0–T1) IL-17α**	1.00	0.99 – 1.00	0.92	0.55 (0.38 – 0.71)
**Δ% (T0–T1) IL-1β**	1.00	0.99 – 1.00	0.09	0.57 (0.42 – 0.72)
**Δ% (T0–T1) IL-2**	1.00	0.99 – 1.00	0.91	0.49 (0.33 – 0.65)
**Δ% (T0–T2) IL-4**	1.00	0.99 – 1.01	0.34	0.56 (0.41 – 0.72)

**Δ% (T0–T1) IL-5**	1.00	0.99 – 1.00	0.50	0.49 (0.32 – 0.65)
**Δ% (T0–T1) IL-7**	0.99	0.99 – 1.00	0.89	0.60 (0.46 – 0.74)
**Δ% (T0–T1) MIP-1α (CCL3)**	1.00	0.99 – 1.00	0.59	0.59 (0.42 – 0.75)
**Δ% (T0–T1) TNFα**	0.99	0.99 – 1.00	0.40	0.59 (0.43 – 0.75)
**Δ% (T0–T1) sPD-L1**	1.00	1.00 – 1.00	0.77	0.51 (0.39 – 0.63)
**Δ% (T0–T2) sPD-L1**	0.99	0.99 – 1.00	0.77	0.51 (0.34 – 0.69)

Alb: albumin; AUC: area under the curve; CAR: CRP-to-albumin ratio; CCL3, 4, 20: C-C motif chemokine ligand; CI: confidence interval; CRP: C-reactive protein; CXCL11: C-X-C motif chemokine ligand 11; CX3CL1: C-X3-C motif chemokine ligand 1; C3/4: complement proteins; C3/C4: C3-to-C4 ratio; dNLR: derived NLR; IFN-γ: interferon-γ; IL: interleukin; ITAC: interferon-inducible T-cell alpha chemoattractant; LDH: lactate dehydrogenase; LIPI: Lung Immune Prognostic Index; LMR: lymphocyte-to-monocyte ratio; Ly: lymphocytes; MIP-3α: macrophage inflammatory protein α; Mono: monocytes; Neu: neutrophils; NLR: neutrophil-to-lymphocyte ratio; PLR: platelet-to-lymphocyte ratio; Plts: platelets; SII: Systemic Inflammation Index; sPD-L1: soluble programmed-death ligand-1; T0: baseline/pretreatment sampling; T1: 21 days (before cycle 2); T2: 3 months; WBCs: white blood cells; Δ%: percentage change.

Table S7. Sensitivity analyses for potential clinical confounders (recent radiotherapy exposure and baseline corticosteroid/immunomodulatory treatment) of significant PBIMs from multivariable prognostic analyses. Univariate models were used for association of baseline levels of PBIMs between patients who had not received radiotherapy or corticosteroids/immunomodulators and clinical outcomes.

**6-month clinical benefit for patients without recent radiotherapy exposure**					
**Parameter**	**AUC**	**95% CI**	**OR**	**95% CI**	**p-value**
CRP	0.80	0.49 – 1.00	0.65	0.34 – 0.98	0.04
C4	0.88	0.68 – 1.00	0.77	0.54 – 0.94	< 0.01
**6-month clinical benefit for patients had not received corticosteroids/immunomodulators**					
**Parameter**	**AUC**	**95% CI**	**OR**	**95% CI**	**p-value**
CRP	0.83	0.64 – 1.00	0.81	0.58 – 1.04	0.10
C4	0.92	0.76 – 1.00	0.77	0.55 – 0.92	< 0.01
**Progression-free survival at 2 years for patients without recent radiotherapy exposure**					
**Parameter**	**C-index**	**95% CI**	**HR**	**95% CI**	**p-value**
CRP	0.45	-0.12 – 1.03	1.27	0.91 – 1.89	0.13
C4	0.66	0.22 – 1.09	1.05	0.89 – 1.22	0.46
IL-6	0.64	0.18 – 1.09	1.95	0.77 – 6.28	0.15
**Progression-free survival at 2 years for patients had not received corticosteroids/immunomodulators**					
**Parameter**	**C-index**	**95% CI**	**HR**	**95% CI**	**p-value**
CRP	0.67	0.28 – 1.05	1.05	0.91 – 1.18	0.46
C4	0.74	0.51 – 0.97	1.03	0.97 – 1.09	0.38
IL-6	0.46	0.03 – 0.88	1.10	0.66 – 1.80	0.70

AUC: area under the curve; CAR: CRP-to-albumin ratio; CI: confidence interval; CRP: C-reactive protein; C3 and 4: complements protein 3 and 4; HR: hazard ratio; IL: interleukin; OR: odds ratio.
